# Genomic insights into antimicrobial resistance and virulence of *E. coli* in central Ethiopia: a one health approach

**DOI:** 10.3389/fmicb.2025.1597580

**Published:** 2025-06-10

**Authors:** Wagaw Sendeku Chekole, Lizel Potgieter, Haileeyesus Adamu, Susanna Sternberg-Lewerin, Tesfaye Sisay Tessema, Ulf Magnusson

**Affiliations:** ^1^Department of Clinical Sciences, Swedish University of Agricultural Sciences (SLU), Uppsala, Sweden; ^2^Institute of Biotechnology, Addis Ababa University, Addis Ababa, Ethiopia; ^3^Institute of Biotechnology, University of Gondar, Gondar, Ethiopia; ^4^Department of Plant Breeding, Swedish University of Agricultural Sciences, Alnarp, Sweden; ^5^Department of Animal Biosciences, Swedish University of Agricultural Sciences, Uppsala, Sweden

**Keywords:** households, *E. coli*, MDR, ARGS, MGEs

## Abstract

Antimicrobial resistance is a global threat causing millions of deaths annually. The study aimed to identify antibiotic resistance genes (ARGs), mobile genetic elements (MGEs), and virulence genes (VGs) and track their dissemination among *E. coli* isolates. Seventy-seven isolates from calves, environments, and human sources were studied. The study involved WGS sequencing, bacterial strains characterized; pan genome, multi-locus sequence typing, and serotyping using O-, and H-typing. The ARGs, VGs, and MGEs were identified using ABRicate against selected respective databases. A maximum likelihood SNP (single nucleotide polymorphism) tree was constructed and visualized with an interactive tree of life (IToL). Descriptive statistics were used to analyze the data. Seventy-seven of the isolates were identified as *E. coli*, later grouped into 5 clades and four known phylogroups. ST10 and O16:H48 were most prevalent in 12 and 42 isolates, respectively. There were about 106 unique ARGs detected between 1.3% and 91.9%, with 57 detected in 40% of isolates. In terms of ARGs, the most common were *bla-ampH* (90.9%), *bla-AmpC1* (89.6%), *tet(A)* (84.4%), *mdf(A)* (81.8%), *aph(3“)-Ib* (79%), *sul2* (79%), *aph(6)-Id* (75%), and *bla-PBP* (70%). It was found that 95 percent (96/106) of ARGs came from at least two sources. The majority of detected ARGs exhibited high concordance between phenotypic resistance and ARGs profiles (JSI ≥ 0.5). In eight isolates, mutations in the *gyrA (3)* and *par-C/E (5)* genes led to ciprofloxacin and nalidixic acid resistance. The most common co-occurrences of ARG and MGE were *Tn3* with *bla-TEM-105* (34), *Int1* with *sul1* (13), and *dhfr7* (11). Meanwhile, the most frequently detected VGs (*n* ≥ 71 isolates) included *elfA-G, fimB-I, hcpA-C, espL, ibeC, entA, fepA-C, ompA, ecpA-E, fepD, fes*, and *ibeB*. Nearly, 88.3% (128/1450) VGs were shared in isolates from at least two sources. ETEC (53.2%), EAEC (22.1%), and STEC (14.3%) were the three most frequently predicted pathotypes. Despite significant ST diversity, ARGs and VGs showed an extensive distribution among the study groups. These findings suggest limited clonal transmission of isolates. In comparison, the wide distribution of ARGs and VGs may be attributed to horizontal gene transfer driven by similar antibiotic selection pressures in the study area.

## 1 Introduction

Antimicrobial resistance (AMR) is a global concern. Multidrug-resistant (MDR) bacterial infections are increasingly undermining both the veterinary and public health sectors. Globally, about 1.27 million deaths were associated with bacterial AMR in 2019 (Murray et al., 2022). Ten million AMR-related deaths are expected annually by 2050, with over 90% of these deaths occurring in Asia and Africa (WHO, 2014; de Kraker et al., 2016).

Additionally, AMR has resulted in a significant financial burden on an international level and is predicted to cause 1–3.4 trillion USD loss by 2030 (World Bank, 2017). In the context of One Health, antibiotic resistance genes (ARGs) are crucial components that circulate among bacteria in humans, animals, and the environment. Soil and wastewater are major reservoirs of antibiotic-resistant bacteria (ARB), as well as antibiotic residues. ARBs and their ARGs can be transferred from the environment to animals and humans (Wellington et al., 2013). Antibiotic use in livestock for treatment, disease prevention, and growth promotion accelerates the emergence and spread of ARGs to humans and the environment (Van et al., 2020). Resistant pathogenic bacteria in the animal gut microbiome may contribute to the dissemination of ARGs across various settings (Amaro et al., 2023).

One of the causes of the rise in AMR is the horizontal gene transfer (HGT) of mobile genetic elements (Partridge et al., 2018). Several genomic structures, including plasmids, insertion sequence elements, transposons, and integrative conjugative and mobilizable elements, facilitate the rapid dissemination of ARGs among bacteria (Carattoli et al., 2014). Plasmid-mediated transmission is one of the main routes for the acquisition of external genetic material, including ARGs, by bacteria (Branger et al., 2018). Moreover, the bacterial genome undergoes rapid mutations in response to a variety of stimuli, some of which lead to the occurrence of resistant bacterial clones (C Reygaert, 2018).

*Escherichia coli*, which belongs to the family Enterobacteriaceae, is known for causing a wide range of infections in both humans and animals. Pathogenic *E. coli* strains have frequently been reported as a cause of disease in children (Snehaa et al., 2021; Ali Mandeel et al., 2022) and calves (Umpiérrez et al., 2021; Chekole et al., 2023) with severe diarrhea, urinary tract infections (UTIs) (Kim et al., 2021), and wound infections (Saeed et al., 2009). In addition, MDR *E. coli* strains are emerging at an alarming rate. The efficacy of modern antimicrobials, which were once considered as the last resort, is starting to decline (Edwards et al., 2022). Globally, the reported median rate of fourth-generation cephalosporin-resistant *E. coli* in 76 countries in 2020 was 42% (WHO, 2022). By 2020, *E. coli* resistant to several antibiotics, including fluoroquinolones, accounted for 20% of UTIs (WHO, 2022).

In Ethiopia, 14.3% Shiga toxin-producing *E. coli O157:H7* in food of bovine origin (Gugsa et al., 2022) and 64% diarrheagenic *E. coli* isolates in diarrheic children and contact calves (Belete et al., 2022) were reported. In addition, 62.7% multidrug-resistant *E. coli* were reported in wastewater (Kebede et al., 2022), and 42.2% in various clinical samples (Bitew, 2020). Many of these studies used PCR while only a limited number of studies have utilized WGS in an attempt to fully characterize *E. coli* (Wolde et al., 2024b). Research involving WGS on *E. coli* ARGs, VGs and transmission across humans, animals, and the environment is rare. The current study aimed to uncover more about how ARGs in *E. coli* spread in rural households in a low-income country. Therefore, this study used WGS to comprehensively characterize ARGs, MGEs, and VGS in MDR *E. coli* isolates obtained from diarrheic calves, in-contact humans, and the farm environment in the same households. Moreover, the genetic relatedness among the isolates was studied to understand the possible transmission of these isolates in the households.

## 2 Materials and methods

### 2.1 Study area and period

**Figure 1:**
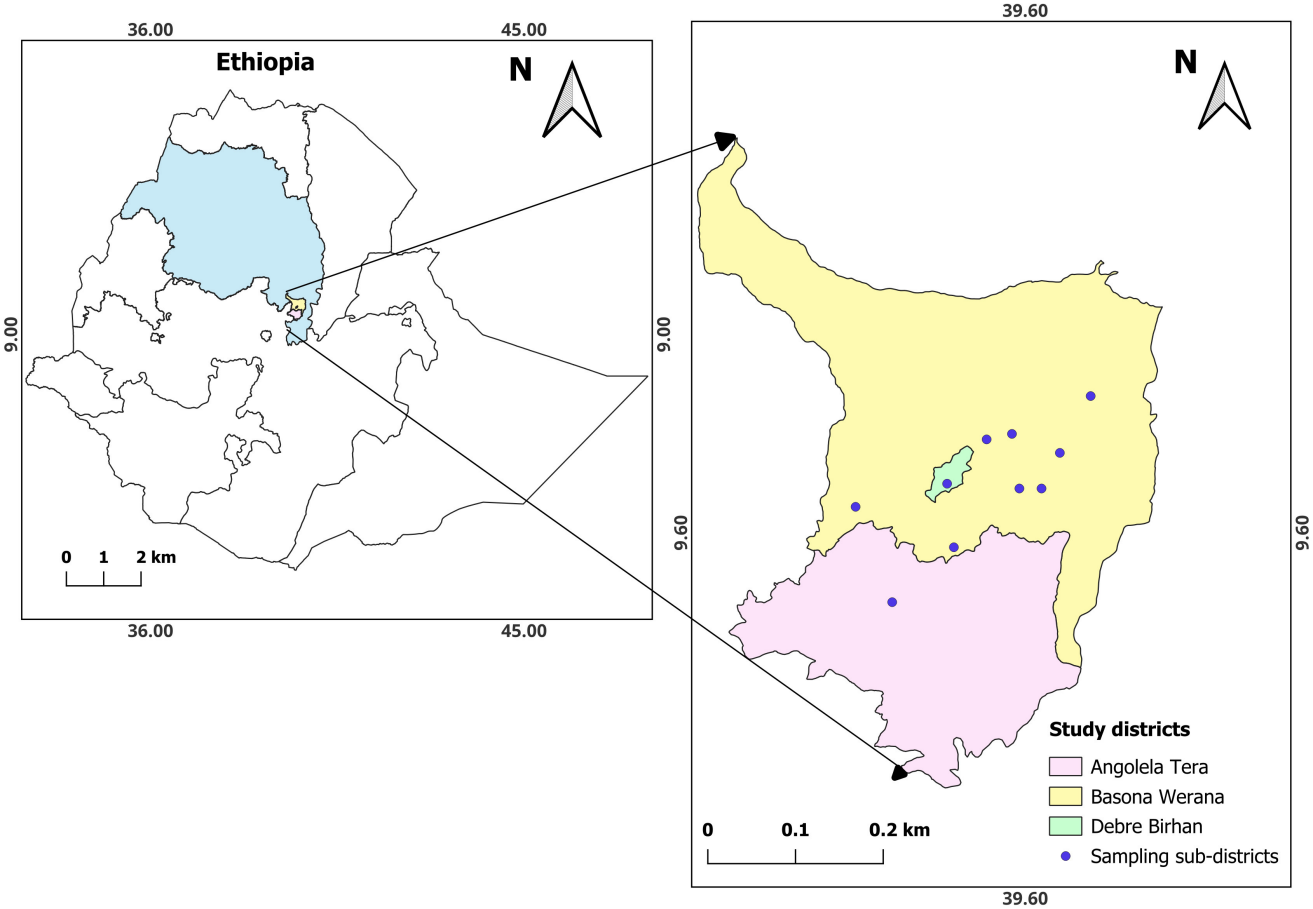
Location of the study area. The study area is located in the top right map and includes ten sampling sub-districts in the two districts. Escherichia coli isolates were obtained from human, calf, and environmental samples collected in the households within the study sub-districts (Chekole et al., 2025).

The study was conducted in two rural districts, Basona Werana and Angolela Tera, located in the Amhara National Regional State (ANRS) in Central Ethiopia (Figure 1). The districts practice a mixed crop-livestock production. The study isolates were obtained from samples collected in 10 sub-districts: 9 in Basona Werana and 1 in Angolela Tera district.

### 2.2 Included *E. coli* isolates

**Figure 2:**
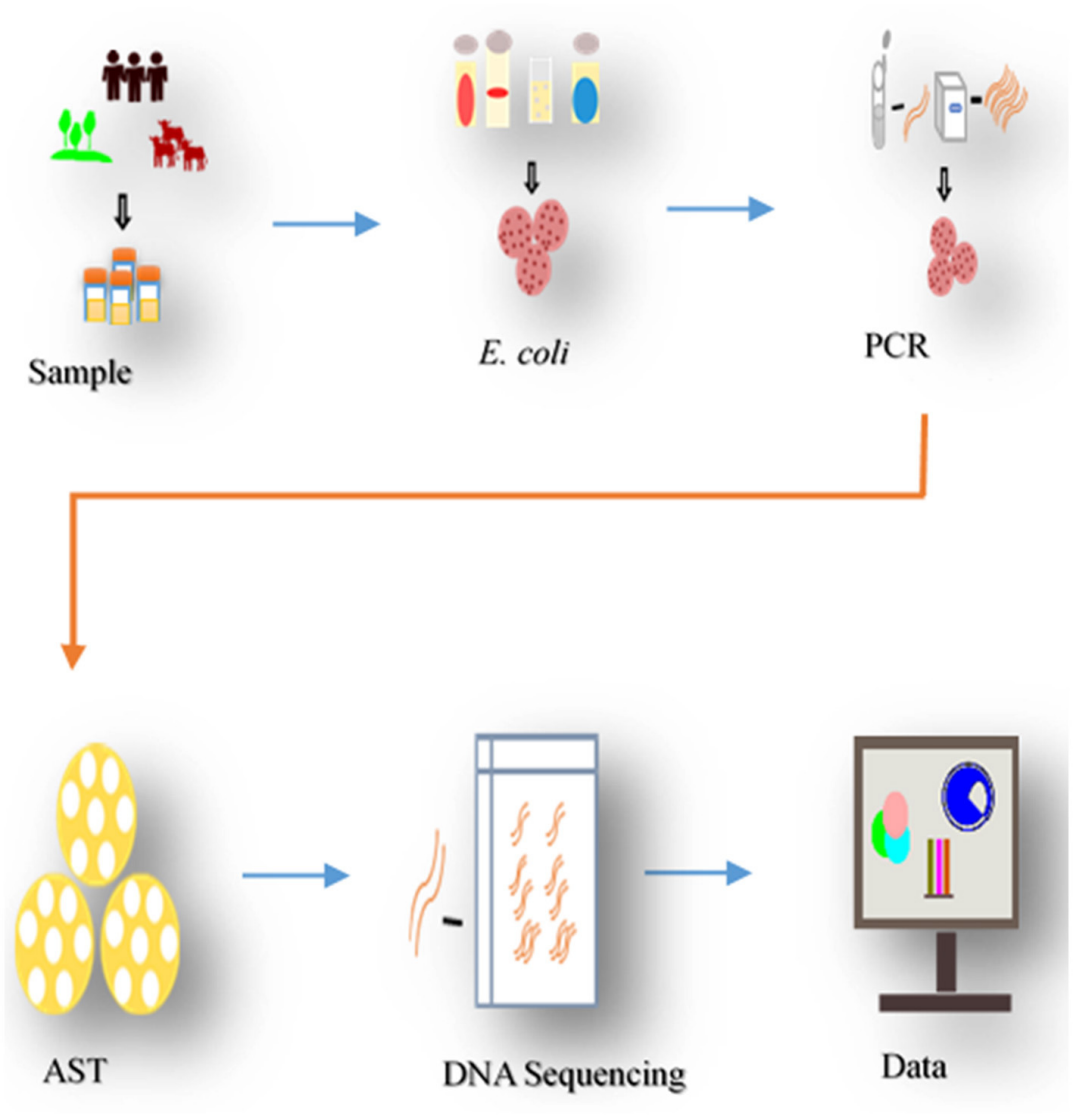
Process of sampling and analysis. The study collected samples from diarrheal calves, environment, and in-contact individuals in the households, isolated E. coli, pathotyped, tested for antibiotic susceptibility (AST). Resistant isolates underwent DNA sequencing to identify ARGs determinants, following bioinformatics analysis.

In this study, *E. coli* isolates of different pathotypes described in previous studies (Chekole et al., 2023, 2025) were further characterized. A total of 78 MDR (resistant to at least 3 antibiotics from different classes) *E. coli* isolates from calves (*n* = 31), environments (*n* = 23), and humans (*n* = 24) were used. The isolates were obtained from concurrently collected from the 98 household samples (Figure 2). After sequential exclusion through biochemical confirmation, pathotyping, and resistance profiling, 78 of isolates were selected from 44 of the households (Chekole et al., 2023, 2025). Households were geographically close, and calf-rearing was common, often in mixed-species settings and isolate selection prioritized broader household coverage over multiple isolates per household.

### 2.3 Whole-genome sequencing

DNA was extracted from the isolates using a bio-basic purification kit (Ontario, Canada), according to the manufacturer's instructions. Subsequently, the isolated DNA was quantified using a Qubit^®^ 3.0 fluorometer (Life Technologies, Malaysia), and purity and integrity were assessed using agarose gel electrophoresis. Sequencing libraries were prepared from samples with ≥100 ng DNA using the TruSeq Nano DNA sample preparation kit and unique dual indices (Illumina, USA) targeting an insert size of 350 bp. Library preparation was performed following the manufacturer's instructions. Pooled DNA libraries were sequenced using the Illumina NovaSeq 6000 system with 150 bp paired-end reads, utilizing an SP flow cell and v1.5 sequencing chemistry (Illumina, USA). The sequencing was carried out at SciLifeLab's SNP&SEQ Technology Platform (Uppsala, Sweden) for genotyping and sequencing.

### 2.4 Bioinformatics analysis

The quality of the raw reads was checked with FastQC v0.11.9 (Andrews, 2010). According to the quality and read coverage analysis (Supplementary Figure 1) raw reads were in high quality and required no trimming. The high-quality reads were *de novo* assembled using SPAdes v3.15.5 (Prjibelski et al., 2020). The quality of the assembled reads was assessed using QUAST v5.0.2 (Gurevich et al., 2013) and BUSCO v5.5.0 (Manni et al., 2021). Species identification and average nucleotide identity (ANI) estimation were performed from assembled draft genomes using GTDB-Tk v2.3.2 (Chaumeil et al., 2022). The assembled reads of *E. coli* confirmed isolates were annotated using Prokka v2.0 (Seemann, 2014), and pan-genome analysis was performed using Roary v3.13.0 (Page et al., 2015). The Achtman seven-locus (*adk, fumC, gyrB, icd, mdh, purA*, and *recA*) scheme multi-sequence type (MLST) for each isolate was identified using the PubMLST database (Jolley et al., 2018). *E. coli* isolates were serotyped by O- and H-typing (Roer et al., 2017), and FimH-typing (Joensen et al., 2015). The MobileElementFinder (https://cge.food.dtu.dk/services/MobileElementFinder/) was used to detect MGEs flanking O-antigen (Johansson et al., 2021). Phylogroups were assigned using Clermont typing and genetic profile classification (Beghain et al., 2018). The probability of isolates being a human pathogen (PoHP) was assessed using PathogenFinder (Cosentino et al., 2013). The draft genomes were used to identify virulence genes using ABRicate v1.0.1 running against the virulence factor database (VFDB), and browsing VFDB online where in both cases default parameters were used (Chen et al., 2016). In addition VirulenceFinder (Malberg Tetzschner et al., 2020) was used to increase the likelihood of detecting virulence specific virulence genes. isolates were pathotyped into their respective groups with the use of ECtyper v2.0.0 (Bessonov et al., 2021) and manual inspection of the detected pathotype-specific virulence genes extracted from VFDB and VirulenceFinder (Chen et al., 2016). ARGs were identified using ABRicate v1.0.1 against four regularly updated databases including; ARG-annot (Gupta et al., 2014), AMRFinderPlus (Feldgarden et al., 2021), ResFinder (Florensa et al., 2022), and CARD (Alcock et al., 2023) with an identity threshold of 95% and minimum sequence coverage of 85%. MGEs such as integrons were identified using IntegronFinder v2.0 (Néron et al., 2022). Plasmid incompatibility groups were identified using PlasmidFinder v2.0.1 (Carattoli et al., 2014) with its own database (version 2024-03-17). The ARGs-MGEs co-occurrence analysis was performed for ARGs and MGEs (plasmids, integrons, and transposons) co-localized on the same contig in the draft genome. The maximum likelihood single nucleotide polymorphism (SNP) tree was constructed using CSI Phylogeny 1.4 (call SNPs and infer phylogeny) of Centre for Genomic Epidemiology (CGE) with a default input parameter: minimum depth at SNP positions; 10, relative depth at SNP positions; 10, minimum distance between SNPs (prune); 10, minimum SNP quality; 30, minimum read mapping quality; 25, minimum Z-score; 1.96 was applied for maximum likelihood phylogeny tree construction (Kaas et al., 2014). For the CSI Phylogeny analysis, the *E. coli K-12 MG1655* reference genome was used. The constructed tree was visualized and annotated using Interactive Tree of Life (iTOL) (Letunic and Bork, 2024).

### 2.5 Data analysis

The detected ARGs from the four ARG databases were harmonized and de-replicated. The harmonized and de-replicated ARGs and VGs in the isolates were initially recorded as binary data: presence (1) or absence (0). Analysis of Variance (ANOVA) was utilized to analyze PoHP among isolate sources, and descriptive statistics were employed to compare the incidence of ARGs and VGs. The previous phenotypic resistance data (Chekole et al., 2025) were used for Jaccard similarity Index (JSI) analysis between phenotypic and genotypic resistance as previously described (Orlek et al., 2023). The study evaluated the concordance between ARGs conferring resistance to specific and multiple drug classes and corresponding phenotypic resistance profiles, with a score of 1 indicating perfect concordance. Similarly, a concordance analysis between PCR and WGS pathotyping was performed using PCR detected pathotypes from previous pathotyping data (Chekole et al., 2023, 2025) and the present WGS-based prediction of pathotypes. In addition, various plots including bar charts were used to visualize the occurrence of ARGs and VGs among isolates in calves, the environment, and humans. The co-occurrence of ARGs and MGEs among the isolates was visualized by a co-occurrence bubble plot. The plots were created using ggplot a versatile plotting package in R, to effectively demonstrate the data (Wickham, 2016).

## 3 Results

### 3.1 Genome characteristics of isolates

**Figure 3:**
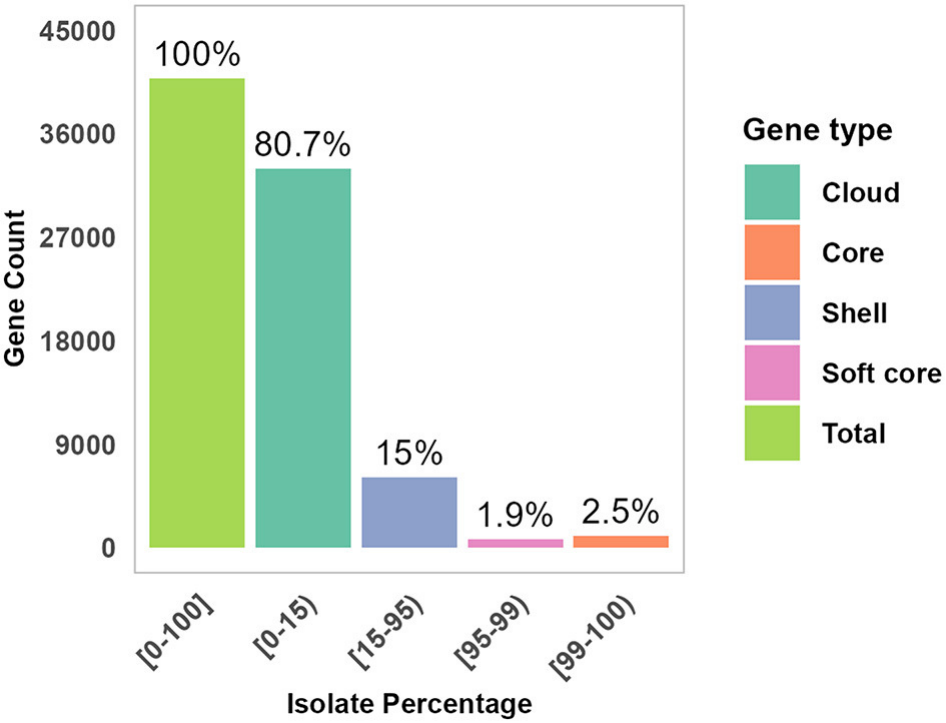
Genomic distribution and features across isolates. The pan-genome distribution across gene categories is presented: core, Soft, Shell, and Cloud proportion of each gene group is shown within the total pan-genome.

The sequenced raw readings were in high quality with a read coverage density plot showing that the majority of reads had coverage ranged 100–200 times (Supplementary Figure 1). Genomic distribution and features across isolates are shown in Supplementary Figures 2, 3. The majority of the assembled draft genomes (71.4%; 55/77) had a size of about 6 megabase pairs (Mb), 50% GC content, and N50 values ranging from 0.2 to 3.5 Mb. The BUSCO analysis revealed high genome completeness across isolates, most at 100%, a few at 99%, and one at 88%. The draft genomes were presented with median values of 5,954 for CDS, 3 for CRISPR, 22 for rRNA, and 108 for tRNA. The assembled draft genomes of the 78 isolates were analyzed for species identification; 77 were confirmed as *E. coli*, while one was identified as a *Klebsiella pneumoniae* isolate and subsequently excluded from this study. The high average nucleotide identity (ANI) values, ranging from 96.4% to 98.3% across the 77 isolates, suggested a high degree of genetic similarity to *E. coli* isolates (Supplementary Figure 4). The pan-genome analysis of the isolates revealed a comprehensive genetic repertoire, described as core genes (*n* = 1,005, 2.5%), soft-core genes (*n* = 789, 1.9%), shell genes (*n* = 6,064, 15%), and cloud genes (*n* = 41,092, 80.7%) (Figure 3). In the pan-genome analysis, the core and soft-core genomes combined contributed approximately 4.4% of the total pan-genome content.

### 3.2 Phylogenetic analysis

**Figure 4:**
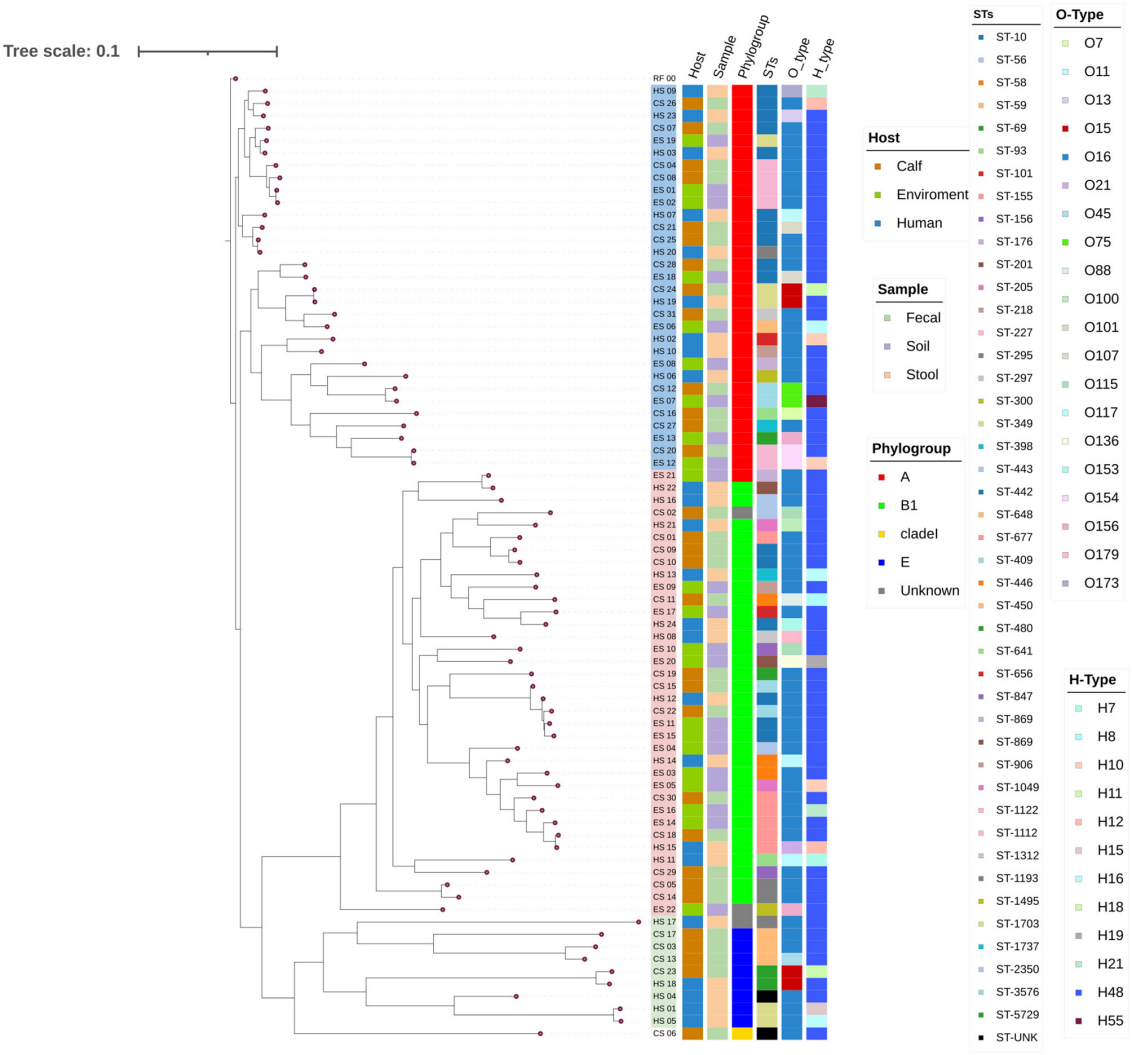
A SNP-based maximum likelihood Phylogenetic Tree. The tree represents the phylogenetic relationships among the different isolates based on the SNP data obtained from isolates using E. coli K-12 MG1655 as a reference genome. The annotations on the tips of the tree provide information about each isolate, including the isolate sources, clade, phylogroup, sequence types (STs), O-types, and H-types. The tree is presented with branch lengths to emphasize the relationships and metadata annotations. Clades A, B, and C are highlighted using light colors (blue, pink, and green, respectively) at the branch tips. RF_00 - *E. coli K-12 MG1655*, CS, Calf sample; ES, Environment sample; HS, Human sample.

The phylogenetic tree was constructed based on 61 core SNPs conserved across all 77 isolates. Pairwise SNP distances ranged from 162 to 45,154, suggesting both closely and distantly related isolates (Supplementary Figure 5). The phylogenetic analysis of the 77 isolates from the three sample sources grouped into 3 main clades: A, B, and C each with distinct characteristics (Figure 4, Supplementary Table 1). Clade B was the largest contained 36 isolates; followed by Clade A contained 31 isolates and clade C had 10 isolates.

Of the 77 isolates, 74 were assigned to the four known phylogroups: 32 isolates assigned to phylogroup A, 33 isolates to phylogroup B1, 8 isolates to phylogroup E, and 1 isolate to clade I (Figure 4, Table 1). Significant genetic diversity was observed in sequence types (STs) with 44 known and 1 unknown, whereas 20 O-types, and 12 H-types were identified. Among the sequence types (STs), the most common included ST10 (12 isolates), ST3576 (5 isolates), ST155 (5 isolates), and ST227 (4 isolates). In the O-types, O16 was represented by 50 isolates, and in the H-types, H48 was represented by 60 isolates, leading to O16:H48 being the dominant serogroup with 42 isolates. Of the 42 O16:H48 isolates, 21 came from calf isolates, 13 came from environment isolates and 8 came from humans (Table 1). In 91% (70/77) of the isolates the detected O-types were found to have the MGE insertion sequence 5 (IS5) (Supplementary Table 1).

**Table 1:** Frequency distribution of sample types, clades, phylogroups, sequence types (STs), O-types, H-types, and serotypes among the 77 *E. coli* isolates.

**Category**	**Sub-category**	**Isolate (N)**
Sample	Calf	31
	Environment	22
	Human	24
Clade	A	31
	B	36
	C	10
Phylogroup	A	32
	B1	33
	Clade I	1
	E	8
	Unknown	3
Sequence types (STs)	ST-10	12
	ST-155	5
	ST-3576	5
	ST-227	4
	ST-(58, 69, 101, 295, 349, 409, 442, 648, 869, 1,703)	2 (each): 2 ^*^10 = 20
	ST-(Others; *n* = 31)	1 (each): 1^*^31 = 31
O-types	O16	50
	O15	4
	(O75, O115, O117, O154, O156)	2 each: 2^*^5 = 10
	O(Others; *n* = 13)	1 each: 1^*^13 = 13
H-types	H48	60
	H10, H16	3 each: 3^*^2 = 6
	H15, H21	3 each: 2^*^2 = 4
	H(Others; *n* = 7)	1 each: 1^*^7 = 7
Serogroup	O16:H48	42
	O16:H16	3
	O115:H48, O15:H48, O156:H48, O16:H10	2 each: 2^*^4= 8
	O:H(Others; *n* = 24)	1 each: 1^*^24 = 24

### 3.3 Antimicrobial resistance genes

**Figure 5:**
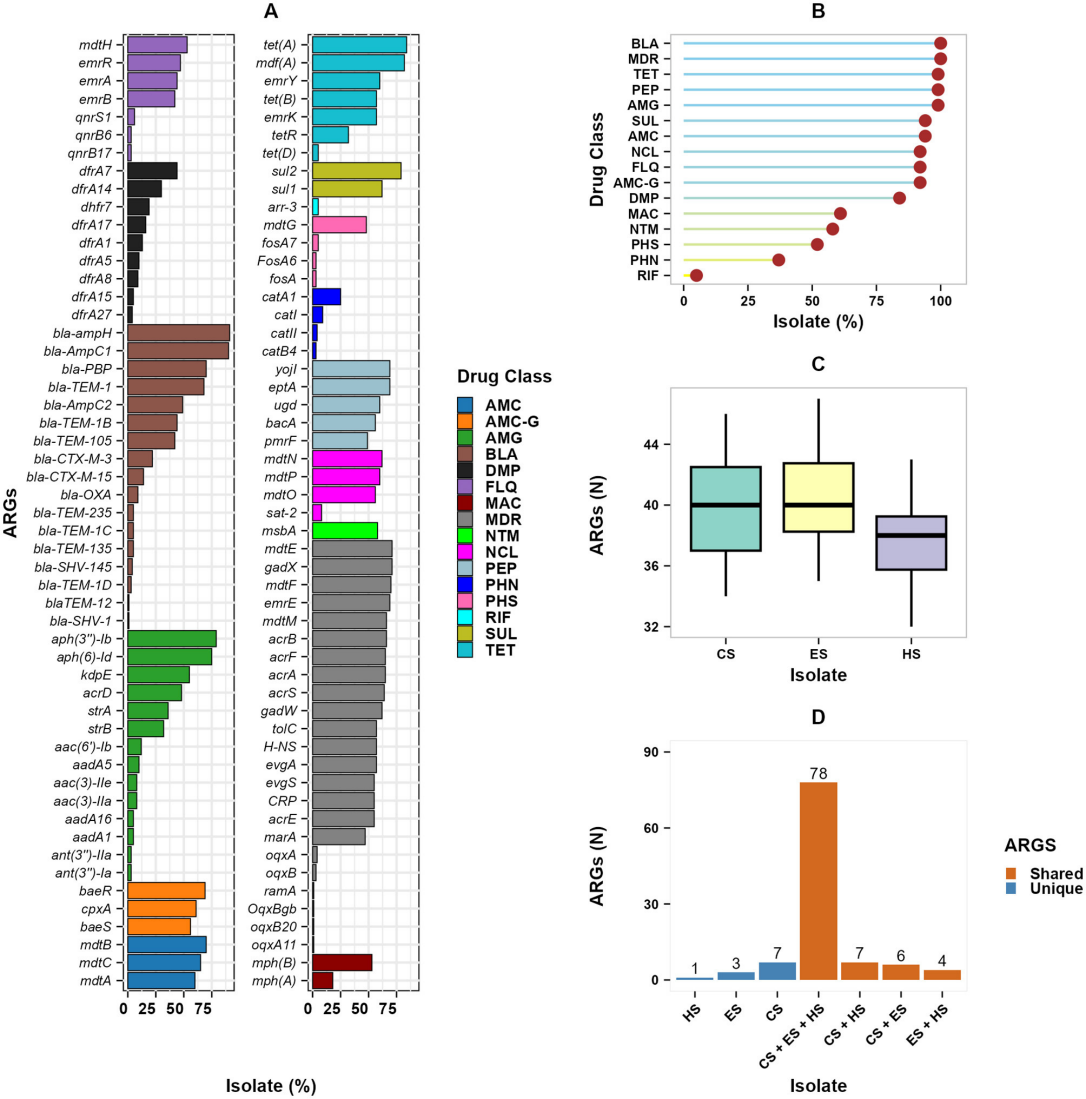
Overall occurrence of ARGs in E. coli isolates. **(A)** The occurrence of each ARG is represented as a percentage in the isolates, grouped based on their associated drug class. The analysis includes 106 unique ARGs, divided into two panels for visualization; **(B)** lollipop plot shows ARGs by drug class; **(C)** boxplot shows the number of ARGs per isolates by sources; and **(D)** the upset plot of ARGs dynamics across isolate sources showing the overlap and uniqueness of ARGs among the studied isolate sources. AMC, Aminocoumarin; AMC-G, Aminocoumarin, Aminoglycoside; AMG, Aminoglycoside; DMP, Diaminopyrimidine; FLQ, Fluoroquinolone; MAC, Macrolide; MDR, Multiple drug class; NCL, Nucleoside; PEP, Peptide; PHN, Phenicols; PHS, Phosphoric acid; RIF, Rifamycin; SUL, Sulphonamide; TET, Tetracycline; BLA, β-lactam. CS, calf samples; ES, environmental samples; HS, human samples.

A total of 106 unique ARGs were identified across all studied isolates (Figure 5A). The occurrence of the various ARGs in the isolates ranged from 1.3% to 90.9%. Fifty-seven of the ARGs were detected in more than 40% of the isolates. The most frequently occurring ARGs were *bla-ampH* (90.9%) and *bla-AmpC1* (89.6%), *tet(A)* (84.4%), and *mdf(A)* (81.8%). Furthermore, among the second most frequently detected ARGs, *mdtB, mdtF, bla_PBP, gadX, mdtE, aph(6)-Id, aph(3”)-Ib*, and *sul2* were detected from 70%−79% of the isolates. In contrast, *oqxA11, ramA, oqxB20, OqxBgb, bla-SHV-1*, and *bla-TEM-12* were less common, detected only in 1.3% of the isolates. The identified ARGs were associated with 15 drug classes. These ARGs conferred resistance to β-Lactam, fluoroquinolone, nucleoside, aminocoumarin, sulphonamide, aminoglycoside, peptide, tetracycline, and multiple drug classes between 92%−100% of the isolates. In contrast, ARGs imparting resistance to rifamycin (5%) and phenicols (37%), exhibited a lower frequency. The occurrence of ARGS differed slightly between the sources of the isolates (Figure 5B). Isolates from calves (CS: 40.27) and the environment (ES: 40.06) exhibited higher mean occurrences of ARGs per isolate than those from human samples (HS: 37.67). Of the 106 ARGs detected, 95 (89.6%) were found in isolates from at least two sample sources; whereas 11 (10.4%) were unique to isolates from one type of sample source (Figure 5C). About 78 (74%) of the ARGs were found in isolates from all the three sample sources (Figure 5D). Additionally, 7 (6.6%) of the ARGs were shared by isolates from calves and humans, 6 (5.7%) between isolates from calves and the environment, and 4 (3.8%) between isolates from humans and the environment. However, 7 ARGs *(fosA7, qnrB17, qnrB6, oqxB, ramA, OqxBgb*, and *SHV-1*) were exclusively detected in calf isolates, 3 ARGs (*oqxA1, oqxB20*, and *TEM-12*) were only found in environmental isolates, and *tet(D)* was only detected from human isolates.

**Figure 6:**
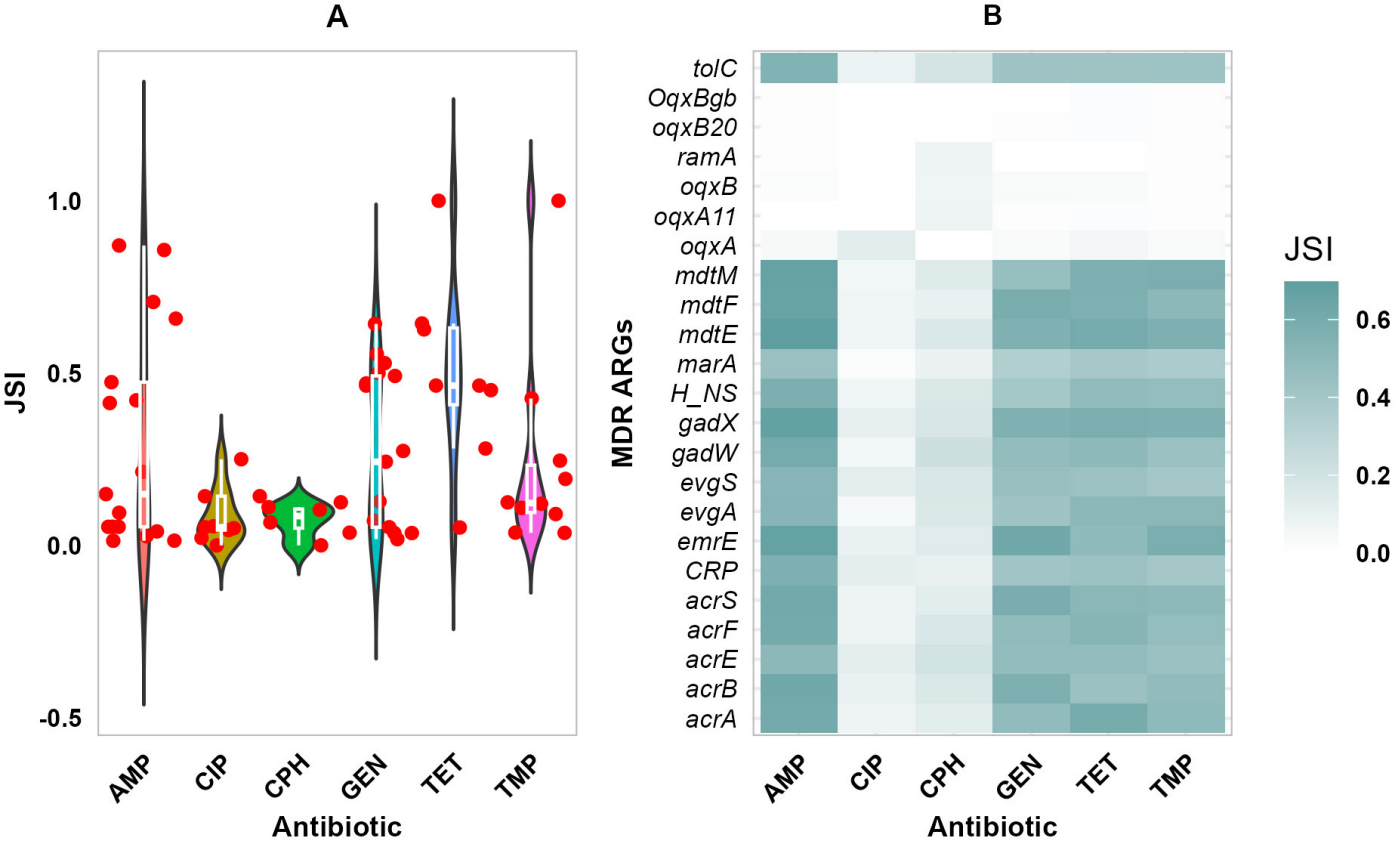
Phenotypic and Genomic resistance concordance (PGRC). The PGRC among E. coli isolates. **(A)** Violin plot illustrating the distribution of JSI for the different antibiotics and ARGs shown in dots. **(B)** Heatmap showing the concordance between phenotypic resistance to multiple antibiotics and ARGs conferring resistance to multiple drug classes, gradient color indicates the extent of concordance. JSI, Jaccard similarity index.

The concordance between phenotypic resistance profiles and the presence of ARGs is shown in Figure 6. The detected ARG profile and the phenotypic resistance profiles showed high concordance (JSI ≥ 0.5). Among ARGs conveying resistance to β-Lactams; *bla_OXA_1, bla_AmpC1, bla_ampH*, and *bla_PBP*, had high concordance to AMP phenotypic resistance shown in Figure 6A (JSI ≥ 0.7). Most isolates were susceptible to CIP and CPH, while a few ARGs conferring resistance to fluoroquinolones and phenicols were detected, with weak concordance. High phenotypic resistance to GEN was highly correlated with *aph(3”)-Ib* and *aph(6)-Id* ARGs both conferring resistance to aminoglycosides (JSI ≥ 0.6), tetracycline phenotypic resistance with *mdf(A)* and *tet(A)* ARGs (JSI ≥ 0.6), and TMP Phenotypic resistance with *dfrA7* ARG (JSI = 0.4). A majority of the ARGs conferring resistance to multiple drug classes had high concordance with AMP, GEN, TET, and TMP (JSI ≥ 0.5), but had low concordance with CIP and CPH (Figure 6B). In addition, ARGs such as *oqxB20, oqxBgb, ramA, oqxB, oqxA*, and *oqxA11* demonstrated weak concordance with all of the phenotypic resistances to the corresponding six antibiotics.

### 3.4 Mobile genetic elements

#### 3.4.1 Plasmids, integrons and transposons

A total of 29 different MGEs were identified, belonging to 3 categories: 27 plasmid amplicon types, one integron (*Int1*) type, and one transposon (*Tn3*) type (Table 2). Of the 27 plasmid amplicon types 20 of them were *IncF* and 5 were *Col* types. The frequency of the plasmid amplicon types ranged from 1.3% to 40.3% of the isolates. The three most prevalent plasmid amplicon types were *IncFIC(FII)* (24.7%), *ColRNAI* (35.1%), and *IncFIB*(*AP001918*) (40.3%). In contrast, *pENTAS02, IncR, IncN, IncHI1B(CIT)*, and *IncFIB(pHCM2)* amplicon types were less frequently appearing in 1.3% of the isolates. Each isolate contained at least one plasmid amplicon type. In addition, the *Int1* integron gene was detected in 44.2% of the isolates, and *Tn3* transposon was found in 51 % of the isolates.

**Table 2:** Distribution of MGEs in *E. coli* isolates (*n* = 77) in smallholder livestock producer households in Ethiopia.

**Category**	**MGEs**	**Isolates *n* (%)**
Plasmid	*Col(BS512)*	6 (7.8)
	*Col(MG828)*	5 (6.5)
	*Col156*	7 (9.1)
	*Col440II*	8 (10.4)
	*Col8282*	10 (13)
	*ColRNAI*	27 (35.1)
	*IncB/O/K/Z*	4 (5.2)
	*IncFIA(HI1)*	3 (3.9)
	*IncFIA*	6 (7.8)
	*IncFIB(AP001918)*	31 (40.3)
	*IncFIB(K)*	5(6.5)
	*IncFIB(pB171)*	7 (9.1)
	*IncFIB(pHCM2)*	1 (1.3)
	*IncFIC(FII)*	19 (24.7)
	*IncFII(29)*	3 (3.9)
	*IncFII(pCoo)*	6 (7.8)
	*IncFII(pHN7A8)*	2 (2.6)
	*IncFII(pRSB107)*	6 (7.8)
	*IncHI1B(CIT)*	1 (1.3)
	*IncI1_Alpha*	8 (10.4)
	*IncI2_Delta*	6 (7.8)
	*IncN*	1 (1.3)
	*IncQ1*	2 (2.6)
	*IncR*	1 (1.3)
	*IncX*	6 (7.8)
	*IncY*	3 (3.9)
	*pENTAS02*	1 (1.3)
Integron	*Int1*	34 (44.2)
Transposon	*Tn3*	39 (50.6)

MGEs, Mobile Genetic Elements; Int1, Integron 1; Tn3, Transposon 3.

### 3.5 Association of ARGs with MGEs

**Figure 7:**
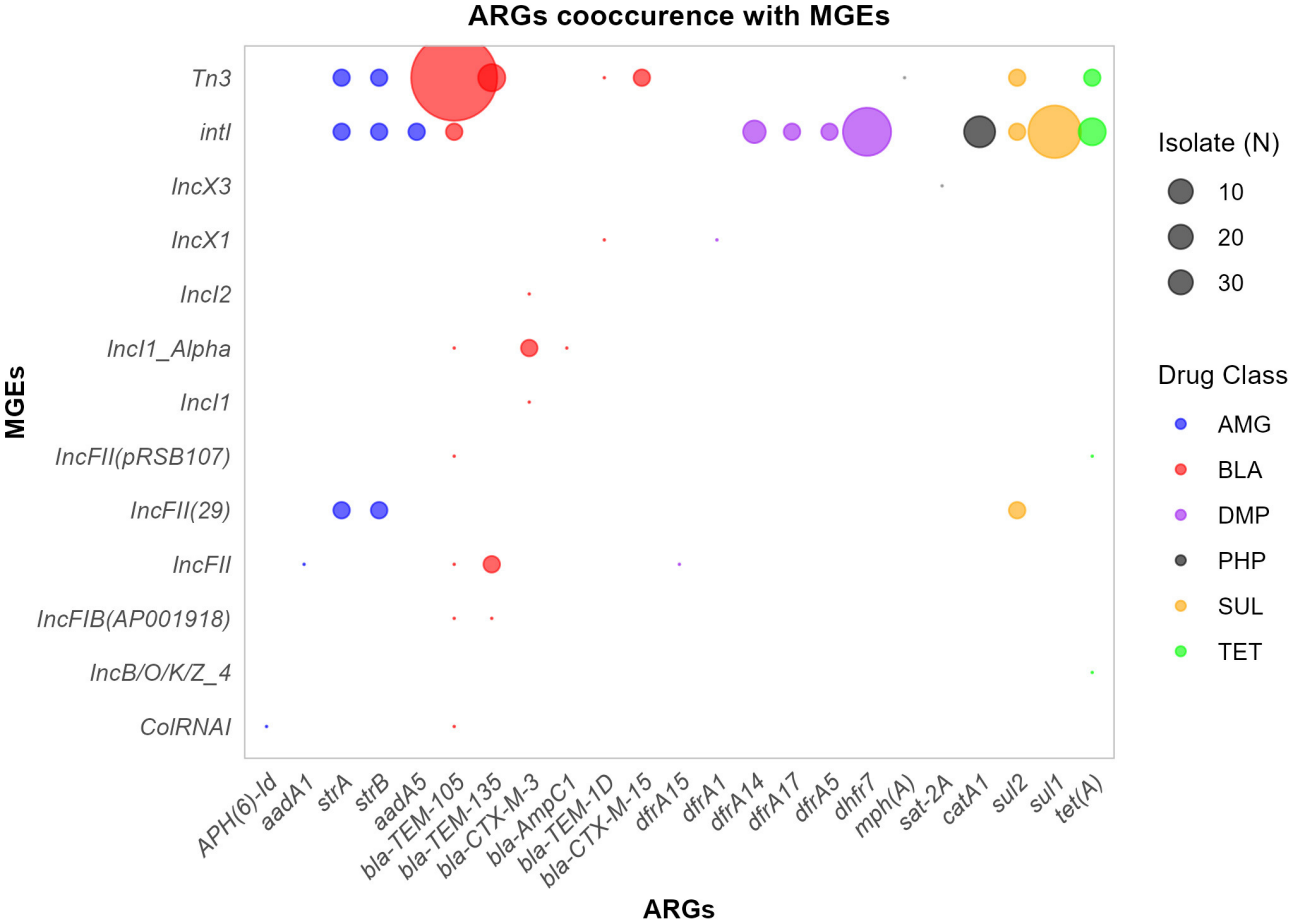
ARGs-MGES co-occurrence. The bubble plot illustrates the frequency of co-occurrence between ARGs and MGEs across isolates, with bubble size proportional to the overall frequency. ARGs are grouped and colored by drug class. AMG, Aminoglycosides; BLA, β-lactams; PHP, Phenicols; TET, tetracyclines; SUL, Sulphonamides; DMP, Diaminopyrimidines.

The distribution of ARGs was examined across the MGEs co-localized within the same contig (Figure 7). The co-localized MGEs were shown to have varying levels of association with the detected ARGs, ranging from 1 to 34 ARGs. The *Tn3* transposon was the most prevalent MGE associated with *bla-TEM-105* co-occurred in 34 isolates. Moreover, the *Int1* integron was found along with 12 different ARGs conferring resistance to aminglycosides (3), β-lactam (1), phenicols (1), Diaminopyrimidine (4), sulphonamide (2), and tetracycline (1) drug classes. *Int1* has most cooccurences with ARGs including *sul1* (13), *dhfr7* (11), *catA1* (5), and *tet(A)* (4) ARGs. Of the 29 plasmids identified, 11 were associated with ARGs. Two (2) co-occurences of ARGs were found in *IncFII(29), IncFII(29), IncFII, IncI1_Alpha*, and *IncFII(29)* plasmids. Overall, of the 29 MGEs detected in isolates, 13 were co-localized with one of the contigs containing ARGs.

### 3.6 Chromosomal mutations and AMR

Chromosomal mutations leading to amino acid changes associated with antimicrobial resistance are shown in Table 3. Among the 77 isolates analyzed, eight isolates were found to have chromosomal mutations associated with resistance: 2 from calves, 1 from the environment, and 5 from humans. The mutations were associated with gyrase *(gyrA*) and topoisomerase (*parC/E*) genes. All these chromosomal changes in these two genes were linked to resistance to the fluoroquinolone drug class; specifically, ciprofloxacin and nalidixic acid.

**Table 3:** Chromosomal mutations found in *gyrA, parC*, and *parE* genes are associated with resistance to drugs in the quinolone/fluoroquinolone drug class in 77 *E. coli* isolates in calves, environment, and human samples from households in Ethiopia.

**Isolate**	**Gene**	**Mutation**	**Nucleotide change**	**Amino acid change**	**Resistance**
CS_03	*gyrA*	p.S83L	TCG -> TTG	S -> L	NAL, CIP
	*gyrA*	p.D87N	GAC -> AAC	D -> N	
	*parC*	p.S80I	AGC -> ATT	S -> I	
	*parE*	p.S458A	TCG -> GCG	S -> A	
CS_17	*parE*	p.I355T	ATC -> ACC	I -> T	
ES_06	*gyrA*	p.S83L	TCG -> TTG	S -> L	
	*gyrA*	p.D87N	GAC -> AAC	D -> N	
	*parC*	p.S80I	AGC -> ATC	S -> I	
	*parE*	p.S458A	TCG -> GCG	S -> A	
HS_01	*parC*	p.S57T	AGC -> ACC	S -> T	
HS_04	*parC*	p.S57T	AGC -> ACC	S -> T	
HS_05	*parC*	p.S57T	AGC -> ACC	S -> T	
HS_17	*gyrA*	p.S83L	TCG -> TTG	S -> L	
	*gyrA*	p.D87N	GAC -> AAC	D -> N	
	*parC*	p.S80I	AGC -> ATC	S -> I	
	*parE*	p.L416F	CTT -> TTT	L -> F	
HS_21	*gyrA*	p.S83L	TCG -> TTG	S -> L	
	*gyrA*	p.D87N	GAC -> AAC	D -> N	
	*parC*	p.S80I	AGC -> ATC	S -> I	
	*parE*	p.S458A	TCG -> GCG	S -> A	

NAL, Nalidixic Acid; CIP, Ciprofloxacin; CS, Calf sample; ES, Environment sample; and HS, Human sample; A, Alanine; D, Aspartic acid; F, Phenylalanine; I, Isoleucine; L, Leucine; N, Asparagine; S, Serine; T, Threonine.

### 3.7 Virulence genes

**Figure 8:**
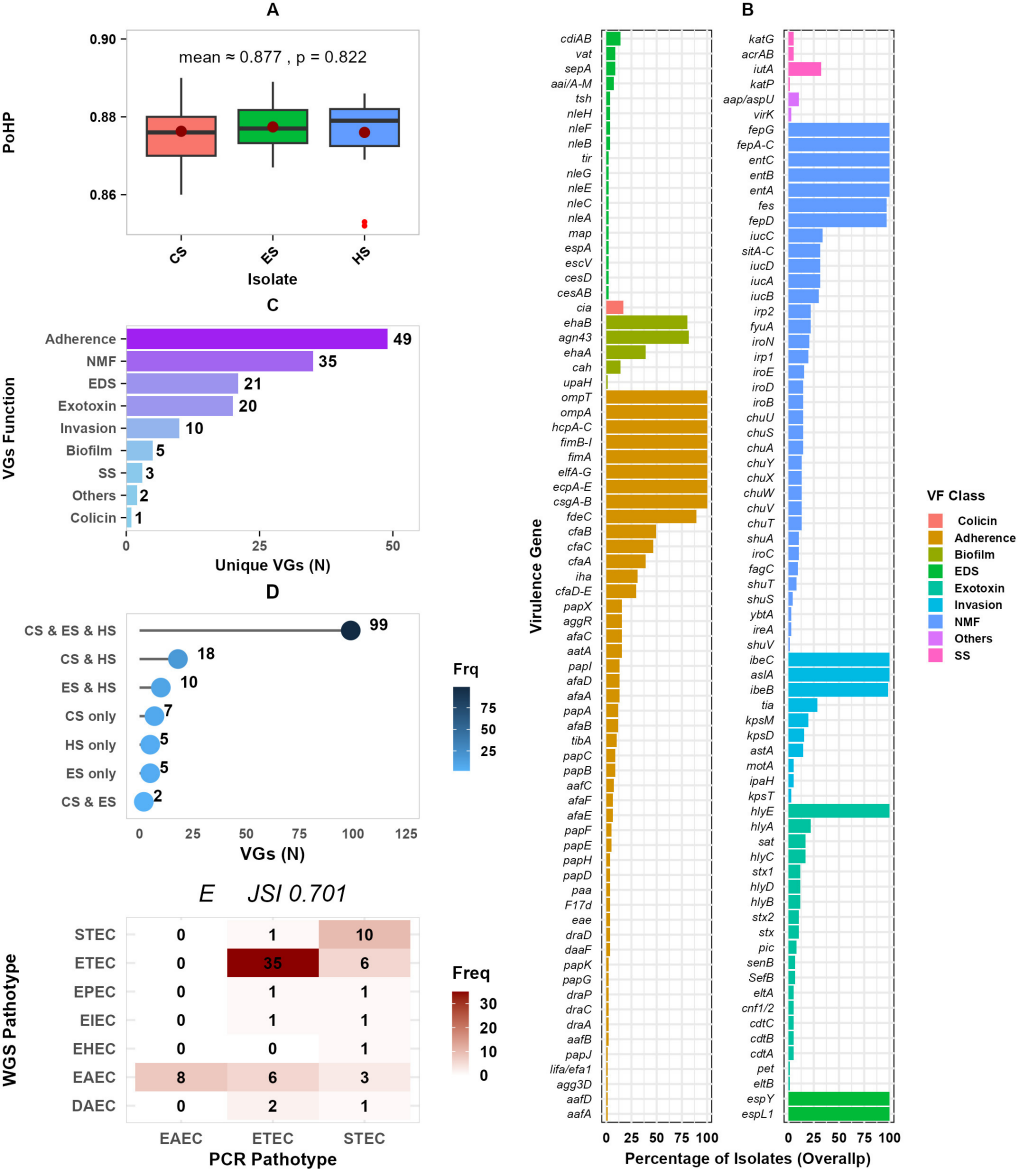
Distribution of VGs by isolate sources and function. **(A)** Presents isolates' the probability of being a human pathogen (PoHP); **(B)** the dot plot presents the percentage of isolates for each VG function, faceted into two panels for better visualization; **(C)** shows distribution of VGs by function; **(D)** presents unique and overlapping of VGs among isolate sources; and **(E)** a concordance frequency heatmap of PCR detected and WGS predicted pathotypes. PoHP, Probability of human pathogen; SS, Stress survival; VGs, Virulence genes; NMF, Nutrition and metabolic factor; EDS, Effector and delivery system; and N, Number. CS, calf samples; ES, environmental samples; HS, human samples.

The selected pathotypes were highly likely to be human pathogens, with a PoHP of 0.88 for each group, with no significant difference between sources (*p* = 0.82) (Figure 8A). About 145 unique VGs with an overall occurrence ranging from 1.3% to 100% among the studied isolate sources were detected (Figure 8B). In all 77 *E. coli* isolates, adherence associated VGs *csgA-B, ecpA-E, elfA-G, fimA, fimB-I, hcpA-C, ompA*, and *ompT* were found. All of the isolates possessed the *espL, hlyE*, and *ibeC* VGs, which are frequently linked to effector and invasion. In addition, nutrition and metabolism factor (NMF) associated *entA, entB, entC, fepA-C*, and *fepG* VGS mainly involved in iron uptake were consistently detected in all isolates. The second most common VGs detected in (*n* ≥ 71) isolates were *ompA, ecpA-E, entB, entC, fepD*, and *fes*, and *ibeB*. Among others, *irp1, irp2, fyuA*, and *sitA-D* implicated in iron and heme transport, *hly* regarded as a known exotoxin, and *agn3* linked to biofilm production were the third most frequently detected VGs. The most prevalent pathotype-specific VGs were *cfaB, cfaC, cfaA, stx, stx1*, and *stx2* detected in 38, 38, 38, 30, 8, 8, and 7 isolates, respectively. Overall, the detected VGs were categorized into nine functional classes, which included colicin, adherence, invasion, biofilm, exotoxins, nutrition and metabolomic factor (NMF), effector and delivery system (EDS), stress survival (SS), and others (Figure 8C). The majority of VGs (105) were classified under adherence (49), NMF (35), and EDS (21). About 128 (88.3%) of the 145 VGs were present in isolates from at least two sources, with 99 (68.3%) of these being identified in isolates from all sources studied (Figure 8D).

The occurrences of predicted pathotypes are shown in Table 4. Enterotoxigenic *E. coli* (ETEC) 41(53.2%), Entroaggregative *E. coli* (EAEC) 17(22.1%), and Shigatoxigenic *E. coli* (STEC) 11(14.3%) were the most frequently detected pathotypes; whereas Enterohemorrhagic *E. coli* (EHEC), Enteropathogenic *E. coli* (EPEC), and *Enteroinvasive E. coli* (EIEC) were less prevalent with 1, 2, and 2 isolates, respectively. Of the 77 pathotypes identified by PCR, 55 (71%, JSI = 0.71) were consistently detected by WGS pathotyping (Figure 8E).

**Table 4:** Distribution of pathotype specific virulence genes among *E. coli* isolates and their classification into pathotype groups.

**Pathotype**	**Virulence gene (s)**	**Frequency (n)**	**Overall frequency – N (%)**
DAEC	*daaF + draD + draP*	1	3(3.9)
	*daaF + draD + draP*	1	
	*daaF + draA + draC + draD + draP*	1	
EAEC	*aap/aspU + aat + aggR + astA*	7	17 (22.1)
	*aatA + astA*	1	
	*aap/aspU + aggR*	1	
	*aatA*	1	
	*astA*	1	
	*aatA + agg3D*	2	
	*aggR + astA*	2	
	*aggR*	2	
EHEC	*eae + stx + stx1 + stx2*	1	1 (1.3)
EIEC	*ipaH + virK*	2	2(2.6)
EPEC	*eae*	2	1 (1.3)
ETEC	*cfaA + cfaB + cfaC + cfaD-E + eltA*	2	41 (53.2)
	*cfaA + cfaB + cfaC + cfaD-E*	13	
	*cfaA + cfaB + cfaC*	12	
	*cfaA + cfaB + cfaC + eltA + eltB*	1	
	*cfaA + cfaB + cfaC + eltA*	1	
	*cfaA*	1	
	*cfaB + cfaC + cfaD-E*	6	
	*cfaB + cfaC*	3	
	*cfaD-E*	2	
STEC	*stx2*	3	11 (14.3)
	*stx + stx1 + stx2*	4	
	*stx + stx1*	4	
**Overall**		**77 (100)**	

## 4 Discussion

In the framework of One Health, this study examined the genome-level analysis of pathogenic *E. coli* isolates, focusing on smallholder livestock production households. It builds on previous works (Chekole et al., 2023, 2025) to provide additional insight into the transmission of antimicrobial-resistant pathogenic *E. coli* among humans, calves, and the environment. This is especially important in the mixed crop-livestock production systems in low-income countries, including Ethiopia where livestock and humans live close to each other and in many cases share the same house.

In the present study, it has been found that the greatest part of isolates had draft genome sizes of about 6 Mb, with a completeness level of 100% and a median of 5,954 CDS. The high BUSCO completeness of the genome indicates the quality and reliability of the genome assemblies in the study. The indicated genome size is higher than the *E. coli K-12* (reference genome, used in this study) with a 4.6 Mb genome size and 4,146 CDS (Dougan et al., 2001). However, the present finding is in agreement with 5.8 Mb and 6,277 CDS in extended-spectrum β-Lactamase (ESBL) producing *E. coli* isolates in Bangladesh (Fatima et al., 2023). There is a similar high, ≥5k CDS counts in some of the pathogenic isolates reported in the same study (Fatima et al., 2023). It has been indicated that a higher genome size is primarily due to the acquisition of different accessory elements (plasmids, pathogenic islands, and prophages) and genes (VGs and ARGs) through HGT (Touchon et al., 2009; Jiao et al., 2024).

The pan-genome analysis showed a large number of stress-resistant cloud genes (80.7%) expected to be shared in only 15% of the isolates. This finding reflects the isolates' genomic plasticity and aligns with suggestions that strain evolution is facilitated by acquiring niche-specific genes while maintaining a stable core genome (Thänert et al., 2022). The SNP-based maximum likelihood clustering of genomes grouped the isolates into three clades, reflecting genomic diversity among *E. coli* isolates. Thirty-three (33) isolates were clustered within phylogroup A, a group described to have originated from commensal lineages (Clermont et al., 2013), some have evolved to pathogenic strains including EAEC and ETEC that can cause life-threatening infections (Awad et al., 2020; Zelelie et al., 2023; Mulu et al., 2024). Serotype O16:H48 (*n* = 42) was the dominant serotype and found mainly in calves (*n* = 21), which indicates its potential reservoir in livestock, and warrants further investigation. This finding is in agreement with reported the predominant (59%) detection of O16:H48 *E. coli* isolates encompassing 7 STs (Duze et al., 2025). The frequent occurrence is also common for other serotype groups, O157 (43.75%) and O26 (37.50%) in STEC isolates (Ranjbar et al., 2018). Despite isolates exhibiting striking sequence type (ST) diversity, serotype O16:H48 predominated in the isolates, which could be associated with evolutionary dynamics. The ST diversity, rooted in mutations and/or recombination of core housekeeping genes, represents long-term lineage divergence (Rocha, 2008). Other researches have highlighted that *E. coli* O-antigen gene clusters (O-AGCs) are 98%−100% identical (Wang et al., 2007), and identical or related with other Enterobacteriaceales (Liu et al., 2014). In addition, insertion sequence 5 (IS5) was found adjacent to the O-antigen gene cluster in 70 of the 77 studied isolates suggesting potential HGT mobilizing this serotype.

The high occurrence of ARGs *bla-ampH, bla-AmpC1, bla-TEM-1, bla-TEM-105, bla-TEM-1B, bla-CTX-M-3*, and *bla-CTX-15* shows the global trends of increased β-Lactam resistance (Edwards et al., 2022; Tohmaz et al., 2022; Wolde et al., 2024a). It has been observed that Ethiopian primary healthcare patients carry high levels of *bla-CTX-M-3, bla-CTX-M-15*, and *bla-TEM-1B* ARGs (Negeri et al., 2023; Wolde et al., 2024a), an indication of increasing resistance to third-generation cephalosporin through time. The predominance of *tet(B), sul1*, of *aph(3”)-Ib* and *aph(6)-Id, dfrA7*, and *dfrA14* confirms the widespread presence of ARGs conveying resistance to many of the present drug classes (Savin et al., 2021; Wolde et al., 2024a). This is particularly concerning given the reliance on trimethoprim-sulphonamides combinations in treating diarrheal diseases in low-resource settings (Cuong et al., 2018). Low occurrence of plasmid-mediated quinolone resistance (PMQR) genes, q*nrB17, qnrB6*, and *qnrS1*, is consistent with isolates from pigs in South Africa (Strasheim et al., 2024). The low number of ARGs from chromosomal mutations (*gyrA/parC*) and PMQR together with the high occurrence of ARGs conferring resistance to multiple drug classes (*acrAB, tolC*, and *mdtH)*, suggests that MDR mechanisms are predominantly responsible for fluoroquinolone resistance in the isolates.

Overall, in this study, the isolates showed a high occurrence of ARGs conferring resistance to β-lactam, aminoglycosides, tetracyclines, peptides, as well as multiple drug classes. It was also noted that the phenotypic resistance and detected ARGs show high concordance. The observed high prevalence of ARGs aligns with our previous findings of high phenotypic resistance to ampicillin, gentamicin, and tetracycline, representing; β-lactam, aminoglycoside, and tetracycline drug classes, respectively (Chekole et al., 2025). The occurrence of ARGs per isolate did not differ significantly between the different sources of the isolates. The slightly higher occurrence of ARGs in isolates from calves and the environment, compared to humans, may be associated with several factors. The sampled calves were active diarrheal cases, which could be subjected to treatment selecting for resistances in the isolates. The high numbers of ARGs in isolates from the environment may be explained by the fact that treated calves excrete drug metabolites into the environment, creating a selective pressure for further resistance development in the fecal contaminated environment. In contrast, the human participants were apparently healthy during sample collection.

Moreover, the study has shown high overlapping of ARGs among isolates from the different sample sources. This could suggest the clonal transmission of isolates among the study sources. However, the ST diversity and cloud gene content are high, suggesting limited clonal transmission and indicating the importance of HGT and selection pressures in acquiring similar ARGs. *E. coli* have high genomic plasticity that allows them to adapt quickly to antibiotic pressures (Jiao et al., 2024). The high prevalence of MDR may be due to inappropriate or prolonged antibiotic use in clinical and agricultural practices in the study area. The findings align with reports of the relevant drug classes being widely used in human healthcare and in animals in Ethiopia (Geta and Kibret, 2021; Tirfe et al., 2023). Inappropriate uses of antimicrobials are common in Ethiopia, both in animal (Bulcha et al., 2024) and human (Erku et al., 2017) settings. In addition, in Ethiopia, it has been indicated that a majority of animals are regularly given antibiotics without a prescription, many farmers often reuse the same drugs for similar symptoms, and antimicrobial sensitivity testing is rarely conducted (Bulcha et al., 2024). Similarly, excessive use of antimicrobials in Nigeria has been linked to increased Salmonella resistance (Jibril et al., 2021), high resistance to multiple drug classes has been linked to inappropriate use in human healthcare systems (Guo et al., 2022).

It was found that *IncF* and *Col* were the most prevalent plasmids. The *IncF* plasmid types are largely involved in carrying ARGs (Villa et al., 2010) and *Col* plasmid types encode colicins and stress survival proteins (Oladeinde et al., 2018). Most plasmids carrying *bla CTX-M* genes were *IncF* types combined with *F-FIA-FIB* (Negeri et al., 2023), indicating multiple plasmid types possessing and transferring ARGs to others. Despite the detection of plasmid replicons such as *IncFIB* and *IncFII* replicons, lack of F-plasmid with intact transfer (tra) genes may limit ARG transfer through conjugation among the study isolates (Tzouvelekis et al., 2012). The isolates were predominantly carrying *Int1* (44.2%) and *Tn3* (51%), consistent with *Int1* being abundant in wastewater isolates (Corno et al., 2023; Tavares et al., 2024). In the present study, *IntI* was found to be frequently linked to *sul1* and *dhfr7*, indicating its role in the spread of ARGs among isolates (Su et al., 2012; Behera et al., 2023). *Tn3* had a high co-occurrence (*n* = 34 isolates) with *bla-TEM-105* ARG consistent with a study involving ESBL isolates primarily carrying the *Tn3* transposon (Gregova and Kmet, 2020). The MGEs-ARGs co-occurrences are considerable, suggesting that MGEs are involved in indirect and bi-directional transfer of ARGs between calves, humans, and the environment.

The study identified 145 different VGs in 9 functional groups. This finding is in line with 103 VGs reported from *E. coli* isolates from humans and pigs in South Africa (Strasheim et al., 2024). The most commonly found adhesins, *ecpA-E, elfA-G, fimB-I*, and *hcpA-C*, are consistent with VGs involved in *E. coli* bacteremia and UTI (Kim et al., 2021). The identification of EDS associated *espL* is in line with what has been reported from bovine sources (Haley et al., 2024) and invasion linked *ibeC* reported from bacteremia and UTI isolates (Kim et al., 2021). The *entA, fepA-C, irp1, irp2, fyuA, iutA*, and *sitA-D* were most commonly found in the isolates confirming the importance of iron uptake for a successful virulence (Kim et al., 2021; Bujnáková et al., 2023). The VG profiles indicate the formation of biofilms and stress survival potentials in the isolates, which are crucial for adaptive strategies. The *Agn43*, a frequently detected VG, is critical in *E. coli* biofilm formation, a trait linked to antibiotic resistance and chronic infections (Michalik et al., 2018). *Cah* and *upaH* are less commonly found in isolates but are associated with urinary tract infections in humans, indicating their niche-specific roles (Kim et al., 2021). The detection of stress survival *acrAB* and *katG* VGs has been linked to efflux-mediated antibiotic resistance (Masi et al., 2017) and oxidative stress response (Jiao et al., 2024).

This study included 7 unique pathotypes, with a predominance of ETEC, characterized by *eltA/eltB* (heat-labile enterotoxins) and colonization factors (*cfaA-D/E*). The present finding is consistent with our previous findings (Chekole et al., 2023, 2025) and other reports from diarrheic calves from Egypt (El-Seedy et al., 2016). All ETEC isolates possessed *cfa* virulence genes, including those co-harboring heat-labile toxins (*eltA* and *eltB*), underscoring the importance of *cfa* in ETEC pathogenesis. EAEC was identified as the second most prevalent pathotype, characterized by *aggR* and *aaiA-M*, which is inconsistent with earlier reports (Chekole et al., 2023, 2025; Strasheim et al., 2024). This dis-concordance in EAEC's epidemiologic investigation is due to its phenotypic definition and lack of a formally recognized genetic definition that complicates its understanding (Boisen et al., 2020). Many of the previous findings targeted *aatA* VG which was not as such indicative. Instead, in a study that redifines the EAEC, *aap* and *aggR* VGs were most frequently linked to EAEC (Boisen et al., 2020). The prediction of STEC marked by shiga-like toxins (*stx1/stx2*) as the third most prevalent pathotype indicates zoonotic risks given its association with severe human diseases (Coccia et al., 2021). The lower frequency of EPEC and DAEC detection aligns with their niche-specific roles in human enteric disease (Snehaa et al., 2021). Additionally, EHEC's low prevalence may be due to a limited cooccurrence of *stx* and *eae* VGs, possibly due to reservoir host variation for these VGs (Nguyen and Sperandio, 2012).

PCR and WGS pathotyping techniques had a high degree of concordance (JSI = 0.71). The results of this study suggest that PCR and WGS are concordant and are reliable techniques for the detection of *E. coli* pathotypes. Comparing both techniques, 29% of pathotypes were differently classified by WGS. PCR pathotyping lacks specificity and sensitivity and is unable to detect all VGs (Akinlabi et al., 2023). The intricacy in big genomic data analysis of the WGS method and lack of a stable pathotyping prediction database can lead to pathotype misassignment (Avershina et al., 2023). WGS, though costly and computationally intensive, is the gold standard in research, epidemiology, and clinical diagnosis (Suminda et al., 2022).

## 5 Conclusions and recommendations

In this study, isolates showed significant diversity in STs with O16:H48 being the most common serotype. There were widespread ARGs and VGs in isolates from calves, humans, and environment, with apparently limited clonal transmission between the study sources. However, there were indications of frequent horizontal transfer of ARGs and VGs among *E. coli* isolates in the study area. For better interventions, it is critical to introduce regular surveillance of pathogens, phenotypic resistance, and ARGs.

## Data Availability

The raw sequence reads used in this study are deposited in the National Center for Biotechnology Information (NCBI) Sequence Read Archive (SRA) under BioProject ID PRJNA1209341 (accessible at: https://dataview.ncbi.nlm.nih.gov/object/PRJNA1209341?reviewer=k23cioqgof59fe37tvqbld8cq6). The BioSample numbers range from SAMN46219280 to SAMN46219356. The data generated in this study can be found in this article or the Supplementary material.
