# Supplementary material for: Genomic insights into antimicrobial resistance and virulence of E. coli in central Ethiopia: a one health approach
Source: Front Microbiol. 2025 Jun 10;16:1597580. doi: 10.3389/fmicb.2025.1597580 (PMC12185407; doi:10.3389/fmicb.2025.1597580)
Supplement: Supplementary file 1 [file Table_1.docx]

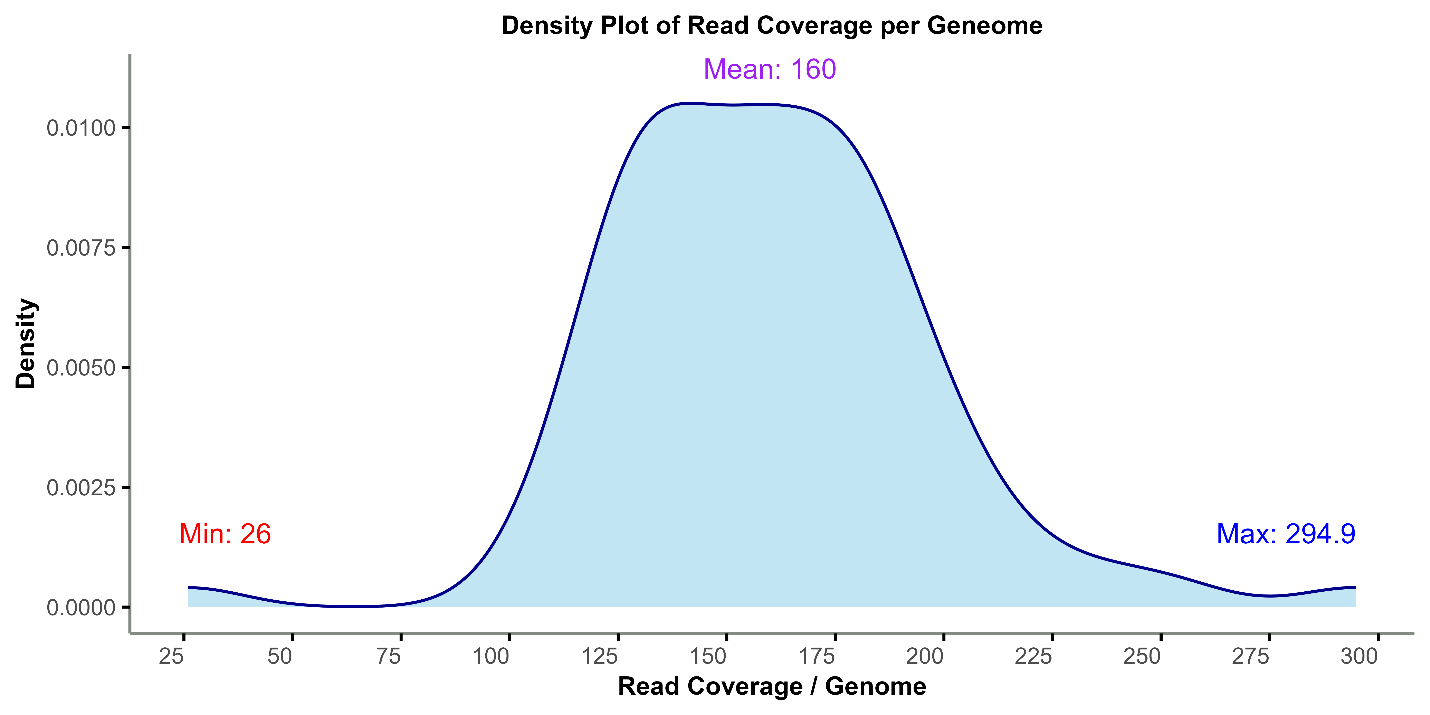


**Figure 1. Density plot of the average read coverage for the sequenced genomes.** The plot highlights the distribution of read coverage, with the minimum (red), mean (purple), and maximum (blue) values. The read coverage per genome was calculated using the formula C=L*N / G, where C is coverage, L is length of reads, N is number of reads, and G is the size of assembled respective genomes.


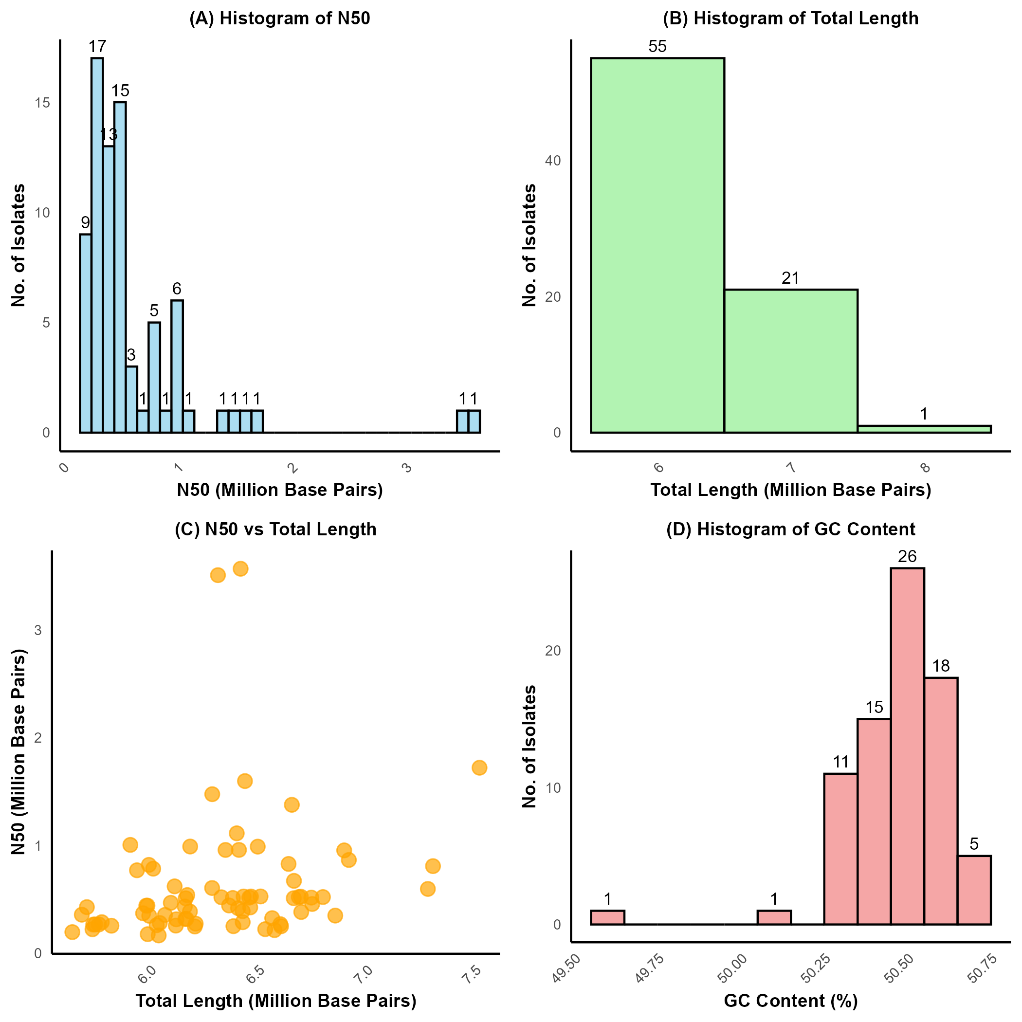


**Figure 2. Quast analysis of assembled draft genomes.** Genomic features of the isolates. The genomic features are illustrated for the isolates, including N50 distribution A, total genome length B, relation of the total genome length with respect to the N50 C, and GC content D. Panel A: The N50 values are a proxy for contiguity in assembled genomes; thus, it depicts the distribution of those values across the isolates. Panel B shows the genome size, represented by the total length of the genomes in million base pairs. Panel C shows the relationship between genome size and N50. It is visualized by a scatter plot. Panel D highlights variation in GC% among the isolates, giving insight into the genomic composition.


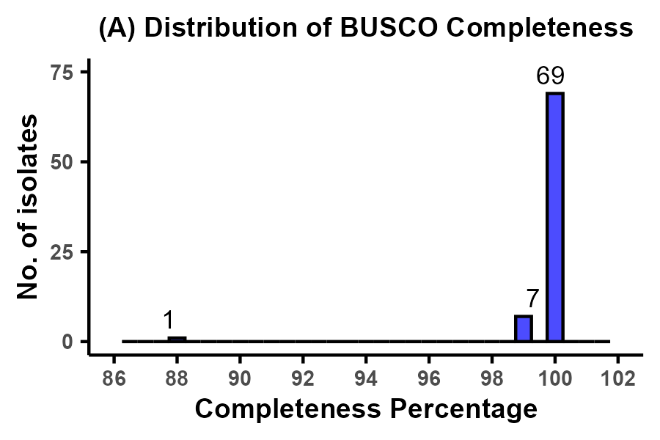

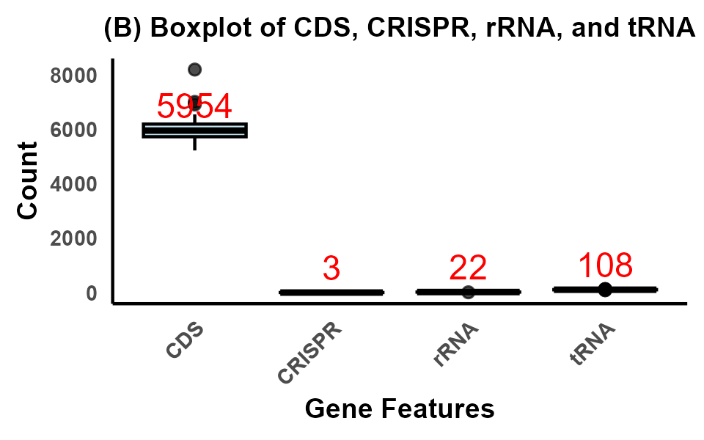


**Figure 3.** **Genomic distribution and features across isolates.** The three plots illustrate key genomic features across the isolates: (A) Histogram showing distribution of BUSCO completeness (%) illustrating the percent of complete single-copy orthologous genes present in each genome; and (B) Boxplot showing the distribution of CDS, CRISPR, rRNA, and tRNA features of the isolates, colored according to the median values in red.


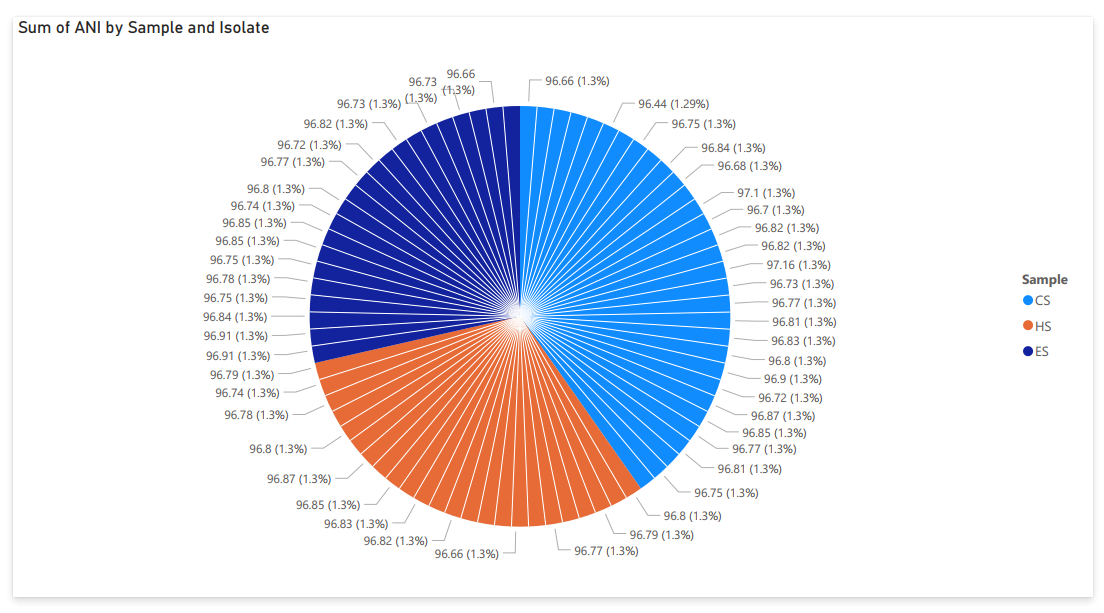


**Figure 4. Average nucleotide Identity (ANI) distribution.** The ANI of the isolates in the three sample sources shown by circular plot found 96% and higher showing isolates are being close to *E. coli* isolate.

**
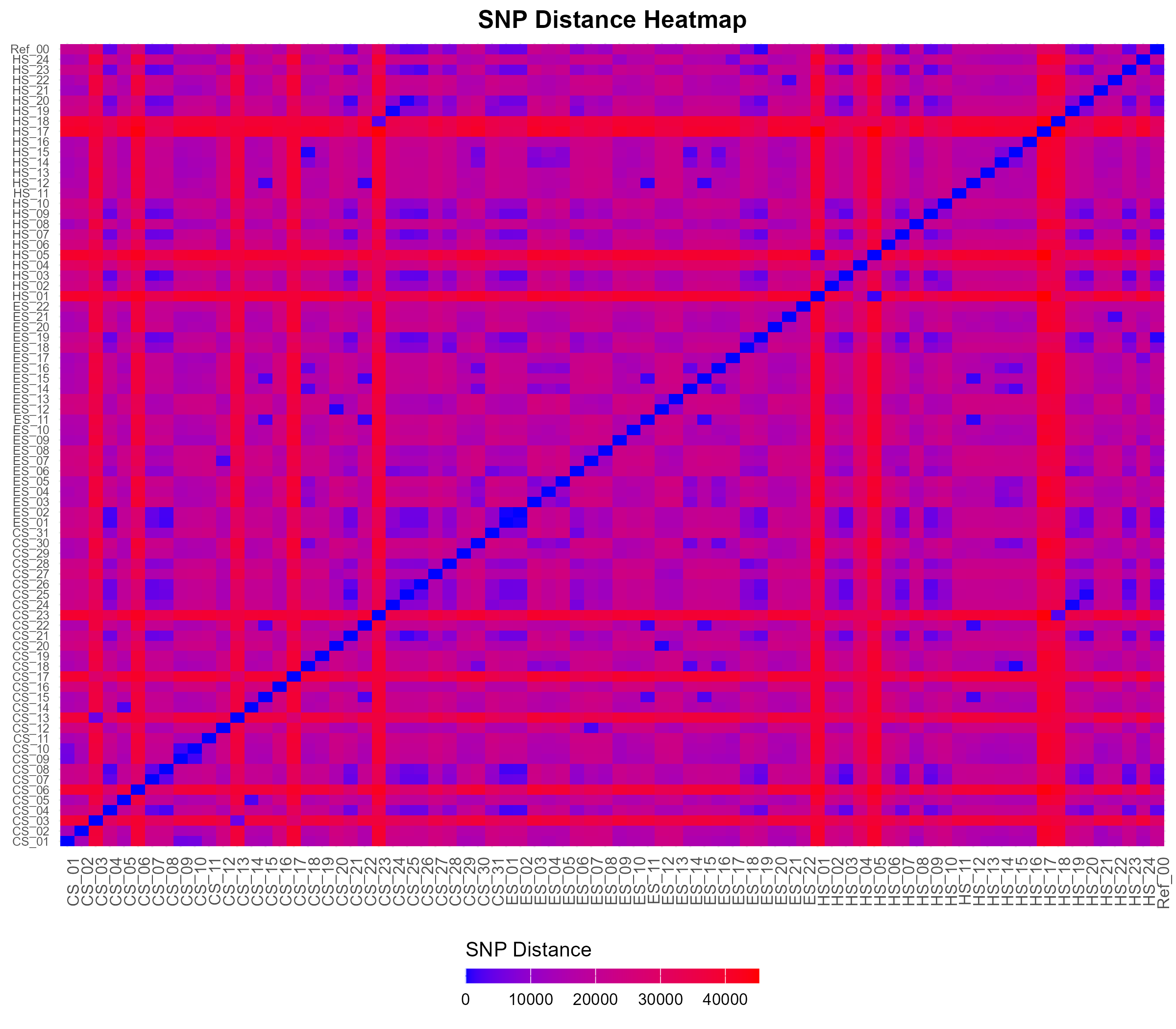
**

**Figure 5. A Heatmap illustrating the pairwise SNP distance between E. coli isolates.** The blue and red colors showing the low and high SNP distance in the isolates. he SNP distance was generated using E. coli K-12 MG1655 as a reference genome, which is also represented in the heatmap as RF_00.

**Table 1. Summary of E. coli isolates showing source sample, phylogenetic group, sequence types (STs), serotypes, detected virulence genes (VGs), and antibiotic resistance genes (ARGs).**

| **Isolate** | **Sample** | **Clade** | **Phylogroup** | **STs** | **O-type** | **H-type** | **Serotypes** | **Virulence Gene(s) - VGs** | **Antibiotic resistance gene(s) - ARGs** | **OAGC - IS** |
| --- | --- | --- | --- | --- | --- | --- | --- | --- | --- | --- |
| CS_01 | Calf | B | B1 | 677 | O16 | H48 | O16:H48 | *aslA, cdiAB, cfaA, cfaB, cfaC, cfaD-E, cia, csgA-B, ecpA-E, ehaA, ehaB, elfA-G, entA, entB, entC, espL1, espY, fdeC, fepA-C, fepD, fepG, fes, fimA, fimB-I, hcpA-C, hlyA, hlyB, hlyC, hlyD, hlyE, ibeB, ibeC, iha, ompA, ompT, stx2, tia* | *acrA, acrB, acrS, aph(3'')-Ib, aph(6)-Id, bacA, bla-AmpC1, bla-ampH, bla-CTX-M-3, bla-PBP, bla-SHV-1, bla-SHV-145, bla-TEM-1, bla-TEM-15, bla-TEM-135, cpxA, CRP, dfrA7, dhfr7, emrE, emrY, eptA, evgS, gadW, gadX, H-NS, mdf(A), mdtA, mdtB, mdtC, mdtE, mdtF, mdtH, mdtN, mdtP, msbA, strA, sul2, tet(A), tolC, ugd, yojI* | *IS5* |
| CS_02 | Calf | B | Unknown | 443 | O115 | H48 | O115:H48 | *aslA, cfaA, cfaB, cfaC, csgA-B, ecpA-E, ehaA, ehaB, elfA-G, entA, entB, entC, espL1, espY, fdeC, fepA-C, fepD, fepG, fes, fimA, fimB-I, hcpA-C, hlyE, ibeB, ibeC, ompA, ompT, tia, aatA, afaA, afaC, afaD, agn43, eltA, eltB, fyuA, irp2, iucC, iutA, pic, sitA-C* | *aac(6')-Ib, acrA, acrB, acrE, acrF, acrS, aph(3'')-Ib, aph(6)-Id, baeR, bla-ampH, bla-CTX-M-3, bla-PBP, bla-TEM-1, catI, cpxA, CRP, emrK, evgA, gadW, gadX, kdpE, mdf(A), mdtA, mdtB, mdtC, mdtP, mph(A), oqxA, pmrF, strB, sul2, tet(A), tet(B), ugd, yojI* | *IS5* |
| CS_03 | Calf | C | E | 648 | O16 | H48 | O16:H48 | *aslA, csgA-B, ecpA-E, ehaB, elfA-G, entA, entB, entC, espL1, espY, fdeC, fepA-C, fepG, fes, fimA, fimB-I, hcpA-C, hlyE, ibeB, ibeC, iha, ompA, ompT, tia, afaA, afaC, afaD, agn43, fyuA, irp2, iucC, iutA, sitA-C, aafC, afaB, afaE, cah, chuA, chuS, chuT, chuU, chuV, chuW, chuX, chuY, daaF, draD, draP, irpirp1, iucA, iucB, iucD, kpsD, kpsM, papA, papB, papI, papX, sat, shuA* | *acrA, acrB, acrE, acrF, acrS, aph(3'')-Ib, aph(6)-Id, bacA, baeR, bla-AmpC2, bla-AmpC1, bla-ampH, bla-TEM-1, bla-TEM-1B, dfrA1, dfrA14, emrA, emrE, emrK, eptA, evgA, gadX, kdpE, mdf(A), mdtA, mdtC, mdtF, mdtH, mdtM, mdtN, mdtO, mdtP, msbA, pmrF, strB, sul2, sul1, tet(B), tolC, ugd* | *IS5* |

| **Isolate** | **Sample** | **Clade** | **Phylogroup** | **STs** | **O-type** | **H-type** | **Serotypes** | **Virulence Gene(s) - VGs** | **Antibiotic resistance gene(s) - ARGs** | **OAGC - IS** |
| --- | --- | --- | --- | --- | --- | --- | --- | --- | --- | --- |
| CS_04 | Calf | A | A | 227 | O16 | H48 | O16:H48 | *aslA, csgA-B, ecpA-E, ehaB, elfA-G, entA, entB, entC, espL1, espY, fdeC, fepA-C, fepD, fepG, fes, fimA, fimB-I, hcpA-C, hlyE, ibeB, ibeC, iha, ompA, ompT, tia, afaA, agn43, fyuA, irp2, iucC, iutA, sitA-C, aafC, afaB, cah, daaF, draD, draP, irpirp1, iucA, iucB, iucD, papI, papX, draA, draC, senB* | *aadA16, acrB, acrD, acrE, acrF, aph(3'')-Ib, aph(6)-Id, baeR, bla-AmpC1, bla-ampH, bla-PBP, bla-TEM-1, bla-TEM-1B, catI, catII, CRP, emrA, emrE, emrK, emrY, evgS, gadX, mdf(A), mdtA, mdtC, mdtE, mdtG, mdtM, mdtN, mdtO, mdtP, mph(A), mph(B), msbA, qnrB6, qnrB17, sul2, sul1, tet(A), yojI* | IS5 |
| CS_05 | Calf | B | B1 | 295 | O16 | H48 | O16:H48 | *aslA, cdiAB, csgA-B, ecpA-E, ehaA, ehaB, elfA-G, entA, entB, entC, espL1, espY, fdeC, fepA-C, fepD, fepG, fes, fimA, fimB-I, hcpA-C, hlyE, ibeB, ibeC, iha, ompA, ompT, stx2, tia* | *aac(6')-Ib, acrA, acrB, acrE, acrS, aph(3'')-Ib, aph(6)-Id, bacA, baeS, bla-AmpC2, bla-AmpC1, bla-ampH, bla-CTX-M-3, bla-PBP, dfrA7, dfrA14, emrB, emrE, emrK, emrR, emrY, eptA, evgS, gadW, gadX, H-NS, kdpE, mdf(A), mdtE, mdtF, mdtG, mdtH, mdtN, mdtO, msbA, pmrF, sul1, tet(A), tolC, yojI* | IS5 |
| CS_06 | Calf | C | cladeI | UNK | O16 | H48 | O16:H48 | *aslA, csgA-B, ecpA-E, ehaB, elfA-G, entA, entB, entC, espL1, espY, fdeC, fepA-C, fepD, fepG, fimA, fimB-I, hcpA-C, hlyE, ibeB, ibeC, ompA, ompT, aatA, agn43, pic, chuA, chuS, chuT, chuU, chuV, chuW, chuX, chuY, kpsD, kpsM, shuA, aai-A/M, aap/aspU, aggR, astA, shuT* | *acrB, acrF, acrS, bacA, baeR, baeS, bla-AmpC1, bla-ampH, bla-CTX-M-3, bla-PBP, bla-TEM-1, CRP, dfrA5, dfrA1, emrA, emrB, emrE, emrK, emrY, eptA, evgA, evgS, gadW, gadX, kdpE, marA, mdtA, mdtB, mdtC, mdtF, mdtH, mdtN, mdtP, msbA, pmrF, strA, strB, sul1, tet(A), tolC, ugd* | IS5 |

| **Isolate** | **Sample** | **Clade** | **Phylogroup** | **STs** | **O-type** | **H-type** | **Serotypes** | **Virulence Gene(s) - VGs** | **Antibiotic resistance gene(s) - ARGs** | **OAGC - IS** |
| --- | --- | --- | --- | --- | --- | --- | --- | --- | --- | --- |
| CS_07 | Calf | A | A | 10 | O16 | H48 | O16:H48 | *aslA, csgA-B, ecpA-E, ehaB, elfA-G, entA, entB, entC, espL1, espY, fdeC, fepA-C, fepD, fepG, fes, fimA, fimB-I, hcpA-C, hlyA, hlyE, ibeB, ibeC, iha, ompA, ompT, agn43, fyuA, irp2, iucC, iutA, irpirp1, iucA, iucB, iucD, acrAB, fagC, ipaH, iroN, katG, motA, papC, virK* | *acrA, acrB, acrS, aph(3'')-Ib, aph(6)-Id, arr-3, bacA, baeR, baeS, bla-AmpC1, bla-ampH, bla-CTX-M-3, bla-TEM-1, bla-TEM-15, bla-TEM-1B, cpxA, dfrA7, dfrA14, emrK, emrY, evgS, fosA7, gadW, gadX, kdpE, mdf(A), mdtA, mdtB, mdtC, mdtE, mdtG, mdtH, mdtN, mdtO, mph(B), OqxBgb, strB, sul2, tet(A), tetR, ugd* | IS5 |
| CS_08 | Calf | A | A | 227 | O16 | H48 | O16:H48 | *aslA, csgA-B, ecpA-E, ehaB, elfA-G, entA, entB, entC, espL1, espY, fdeC, fepA-C, fepD, fepG, fes, fimA, fimB-I, hcpA-C, hlyA, hlyE, ibeB, ibeC, iha, ompA, ompT, agn43, iucC, iutA, iucA, iucB, iucD, acrAB, fagC, ipaH, iroN, katG, motA, papC, virK, iroB* | *acrA, acrB, acrF, aph(3'')-Ib, aph(6)-Id, bacA, baeR, baeS, bla-AmpC1, bla-ampH, bla-TEM-1, bla-TEM-15, bla-TEM-1B, cpxA, dfrA8, dfrA14, dfrA17, dhfr7, emrA, emrB, emrK, emrR, eptA, evgA, fosA7, gadX, H-NS, mdf(A), mdtA, mdtC, mdtE, mdtF, mdtH, mdtM, mdtN, mdtO, mdtP, msbA, qnrS1, sat-2, sul1, tet(A), tet(B), tolC, ugd* |  |
| CS_09 | Calf | B | B1 | 442 | O16 | H48 | O16:H48 | *aslA, cfaA, cfaB, cfaC, cfaD-E, cia, csgA-B, ecpA-E, ehaA, ehaB, elfA-G, entA, entB, entC, espL1, espY, fdeC, fepA-C, fepD, fepG, fes, fimA, fimB-I, hcpA-C, hlyA, hlyE, ibeB, ibeC, ompA, ompT, agn43, iucC, iutA, sitA-C, iucA, iucB, iucD, acrAB, fagC, ipaH, iroN, katG, motA, papC, iroB, iroC, iroD, iroE* | *aadA5, acrA, acrB, acrD, acrE, acrS, aph(6)-Id, baeR, baeS, bla-AmpC2, bla-AmpC1, bla-ampH, bla-TEM-1, bla-TEM-15, bla-TEM-1B, cpxA, dhfr7, emrE, emrK, emrR, emrY, eptA, evgS, gadW, gadX, H-NS, mdf(A), mdtA, mdtB, mdtE, mdtF, mdtM, mdtN, mdtO, mdtP, mph(A), pmrF, strA, sul2, sul1, tet(B), tolC, ugd, yojI* | IS5 |

| **Isolate** | **Sample** | **Clade** | **Phylogroup** | **STs** | **O-type** | **H-type** | **Serotypes** | **Virulence Gene(s) - VGs** | **Antibiotic resistance gene(s) - ARGs** | **OAGC - IS** |
| --- | --- | --- | --- | --- | --- | --- | --- | --- | --- | --- |
| CS_10 | Calf | B | B1 | 442 | O16 | H48 | O16:H48 | *aslA, cfaA, cfaB, cfaC, cfaD-E, cia, csgA-B, ecpA-E, ehaA, ehaB, elfA-G, entA, entB, entC, espL1, espY, fdeC, fepA-C, fepD, fepG, fes, fimA, fimB-I, hcpA-C, hlyA, hlyE, ibeB, ibeC, ompA, ompT, agn43, iucC, iutA, sitA-C, iucA, iucB, iucD, acrAB, fagC, ipaH, iroN, katG, motA, papC, iroB, iroC, iroD, iroE* | *aadA5, acrA, acrB, acrD, acrE, acrS, aph(6)-Id, baeR, baeS, bla-AmpC2, bla-AmpC1, bla-ampH, bla-TEM-1, bla-TEM-15, bla-TEM-1B, cpxA, dhfr7, emrE, emrK, emrR, emrY, eptA, evgS, gadW, gadX, H-NS, mdf(A), mdtA, mdtB, mdtE, mdtF, mdtM, mdtN, mdtO, mdtP, mph(A), pmrF, strA, sul2, sul1, tet(B), tolC, ugd, yojI* | *IS5* |
| CS_11 | Calf | B | B1 | 446 | O88 | H8 | O88:H8 | *aslA, cdiAB, cfaA, cfaB, cfaC, csgA-B, ecpA-E, ehaA, elfA-G, entA, entB, entC, espL1, espY, fdeC, fepA-C, fepD, fepG, fes, fimA, fimB-I, hcpA-C, hlyA, hlyC, hlyE, ibeB, ibeC, ompA, ompT, tia, iucC, iutA, iucA, iucB, iucD, cdtA, cdtB, cdtC, cnfcnf1-2-2, FF17d7d, tibA* | *aac(3)-IIa, aadA1, aadA16, acrE, acrF, aph(6)-Id, baeR, baeS, bla-AmpC1, bla-ampH, bla-PBP, bla-TEM-1, cpxA, dfrA5, dfrA7, dfrA8, emrB, eptA, gadW, gadX, kdpE, marA, mdf(A), mdtA, mdtB, mdtC, mdtF, mdtG, mdtH, mdtM, mdtO, mph(B), pmrF, strB, sul2, tet(A), ugd* | *IS5* |
| CS_12 | Calf | A | A | 409 | O75 | H48 | O75:H48 | *aslA, cfaD-E, csgA-B, ecpA-E, ehaB, elfA-G, entA, entB, entC, espL1, espY, fepA-C, fepD, fepG, fes, fimA, fimB-I, hcpA-C, hlyE, ibeB, ibeC, ompA, ompT, agn43, sitA-C* | *aadA5, aadA1, acrA, acrD, acrF, acrS, aph(3'')-Ib, baeR, baeS, bla-AmpC1, bla-ampH, bla-CTX-M-3, bla-CTX-M-15, bla-PBP, bla-TEM-15, bla-TEM-1B, catA1, cpxA, dfrA5, dfrA1, emrA, emrR, emrY, gadW, kdpE, marA, mdf(A), mdtA, mdtB, mdtC, mdtE, mdtF, mdtH, mdtM, mdtN, mdtP, mph(B), pmrF, sul2, sul1, tet(B), ugd, yojI* | *IS5* |

| **Isolate** | **Sample** | **Clade** | **Phylogroup** | **STs** | **O-type** | **H-type** | **Serotypes** | **Virulence Gene(s) - VGs** | **Antibiotic resistance gene(s) - ARGs** | **OAGC - IS** |
| --- | --- | --- | --- | --- | --- | --- | --- | --- | --- | --- |
| CS_13 | Calf | C | E | 648 | O45 | H48 | O45:H48 | *aslA, cfaB, cfaC, csgA-B, ecpA-E, ehaB, elfA-G, entA, entB, entC, espL1, espY, fdeC, fepA-C, fepG, fes, fimA, fimB-I, hcpA-C, hlyE, ibeB, ibeC, iha, ompA, ompT, tia, agn43, iucC, iutA, sitA-C, cah, chuA, chuS, chuT, chuU, chuV, chuW, chuX, chuY, iucA, iucB, iucD, kpsD, kpsM, papA, papB, papI, papX, sat, shuA* | *acrA, acrB, acrE, acrF, acrS, aph(3'')-Ib, aph(6)-Id, baeR, baeS, bla-AmpC1, bla-ampH, bla-PBP, bla-TEM-1, bla-TEM-15, catB4, cpxA, CRP, dfrA14, emrK, emrY, evgA, gadW, gadX, kdpE, mdf(A), mdtA, mdtB, mdtC, mdtE, mdtF, mdtG, mdtM, mdtO, mph(A), strA, sul2, sul1, tet(A), tet(B), tetR, tolC, ugd, yojI* | *IS5* |
| CS_14 | Calf | B | B1 | 295 | O16 | H48 | O16:H48 | *aslA, csgA-B, ecpA-E, ehaA, ehaB, elfA-G, entA, entB, entC, espL1, espY, fdeC, fepA-C, fepD, fepG, fes, fimA, fimB-I, hcpA-C, hlyE, ibeB, ibeC, iha, ompA, ompT, stx2, tia, agn43, stx, stx1* | *acrB, acrE, acrF, aph(3'')-Ib, aph(6)-Id, bla-AmpC2, bla-AmpC1, bla-ampH, bla-CTX-M-15, bla-PBP, bla-TEM-1B, catA1, dfrA14, emrA, emrE, emrK, emrR, eptA, evgA, evgS, gadW, gadX, H-NS, kdpE, mdf(A), mdtF, mdtG, mdtH, mdtN, mdtO, mdtP, mph(B), msbA, pmrF, sul2, tet(A), tetR* | *IS5* |
| CS_15 | Calf | B | B1 | 3576 | O16 | H48 | O16:H48 | *aslA, cfaA, cfaB, cfaC, csgA-B, ecpA-E, ehaB, elfA-G, entA, entB, entC, espL1, espY, fdeC, fepA-C, fepD, fepG, fes, fimA, fimB-I, hcpA-C, hlyE, ibeB, ibeC, ompA, ompT, agn43, cah* | *acrB, acrD, acrE, acrS, ant(3'')-IIa, aph(3'')-Ib, aph(6)-Id, baeR, bla-AmpC2, bla-AmpC1, bla-ampH, bla-TEM-235, bla-TEM-1, bla-TEM-135, bla-TEM-1B, bla-TEM-1C, catI, cpxA, dfrA7, emrE, emrK, emrY, eptA, evgS, gadX, H-NS, kdpE, mdf(A), mdtA, mdtB, mdtG, mdtM, mdtO, mdtP, mph(A), mph(B), pmrF, sul1, tet(A), tet(B), ugd, yojI* | *IS5* |

| **Isolate** | **Sample** | **Clade** | **Phylogroup** | **STs** | **O-type** | **H-type** | **Serotypes** | **Virulence Gene(s) - VGs** | **Antibiotic resistance gene(s) - ARGs** | **OAGC - IS** |
| --- | --- | --- | --- | --- | --- | --- | --- | --- | --- | --- |
| CS_16 | Calf | A | A | 93 | O7 | H48 | O7:H48 | *aslA, cfaB, cia, csgA-B, ecpA-E, ehaB, elfA-G, entA, entB, entC, espL1, espY, fdeC, fepA-C, fepD, fepG, fes, fimA, fimB-I, hcpA-C, hlyE, ibeB, ibeC, ompA, ompT, aatA, iucC, iutA, sitA-C, cah, iucA, iucB, iucD, kpsD, kpsM, papX, astA, iroN, iroB, iroC, iroD, iroE* | *acrA, acrB, acrE, acrF, aph(3'')-Ib, aph(6)-Id, bla-AmpC1, bla-ampH, bla-TEM-235, bla-TEM-1, bla-TEM-1B, CRP, dfrA7, dfrA14, dhfr7, emrE, emrK, emrR, emrY, evgS, gadW, marA, mdtA, mdtC, mdtE, mdtG, mdtN, mdtP, mph(B), qnrS1, strA, sul2, sul1, tet(A), tolC, ugd, yojI* | *IS5* |
| CS_17 | Calf | C | E | 59 | O16 | H48 | O16:H48 | *aslA, cfaB, cfaC, csgA-B, ecpA-E, ehaB, elfA-G, entA, entB, entC, espL1, espY, fdeC, fepA-C, fepD, fepG, fes, fimA, fimB-I, hcpA-C, hlyE, ibeB, ibeC, iha, ompA, ompT, agn43, fyuA, irp2, iucC, iutA, sitA-C, chuA, chuS, chuT, chuU, chuV, chuW, chuX, chuY, iucA, iucB, iucD, kpsD, kpsM, papI, papX, sat, senB, kpsT* | *acrA, acrB, acrF, aph(3'')-Ib, baeR, baeS, bla-AmpC2, bla-AmpC1, bla-ampH, bla-PBP, bla-TEM-1, cpxA, CRP, dfrA5, dfrA8, emrB, emrE, emrR, emrY, evgS, gadW, gadX, kdpE, marA, mdf(A), mdtB, mdtE, mdtH, mdtM, sat-2, strB, sul1, tet(A), tet(B)* | *IS5* |
| CS_18 | Calf | B | B1 | 155 | O16 | H48 | O16:H48 | *aslA, csgA-B, ecpA-E, ehaA, ehaB, elfA-G, entA, entB, entC, espL1, espY, fdeC, fepA-C, fepD, fepG, fes, fimA, fimB-I, hcpA-C, hlyE, ibeB, ibeC, ompA, ompT, aatA, sat, aap/aspU, aggR, astA, SefB, sepA, vat* | *aac(6')-Ib, aadA5, acrE, acrF, acrS, aph(3'')-Ib, aph(6)-Id, baeS, bla-AmpC1, bla-ampH, bla-PBP, bla-TEM-1, bla-TEM-15, catA1, CRP, dfrA14, emrE, eptA, evgA, evgS, gadW, gadX, H-NS, mdtE, mdtH, mdtM, mdtN, mdtO, mdtP, mph(A), mph(B), msbA, strA, strB, sul2, sul1, tet(A), tet(B), tetR, tolC, yojI* | *IS5* |

| **Isolate** | **Sample** | **Clade** | **Phylogroup** | **STs** | **O-type** | **H-type** | **Serotypes** | **Virulence Gene(s) - VGs** | **Antibiotic resistance gene(s) - ARGs** | **OAGC - IS** |
| --- | --- | --- | --- | --- | --- | --- | --- | --- | --- | --- |
| CS_19 | Calf | B | B1 | 5729 | O16 | H48 | O16:H48 | *aslA, cdiAB, cia, csgA-B, ecpA-E, ehaA, elfA-G, entA, entB, entC, espL1, espY, fdeC, fepA-C, fepD, fepG, fes, fimA, fimB-I, hcpA-C, hlyA, hlyB, hlyC, hlyD, hlyE, ibeB, ibeC, iha, ompA, ompT, stx2, agn43, stx, stx1, tsh* | *aac(6')-Ib, acrD, acrF, aph(3'')-Ib, bacA, baeR, bla-AmpC2, bla-AmpC1, bla-ampH, bla-PBP, bla-TEM-15, bla-TEM-1B, cpxA, CRP, dfrA7, emrA, emrB, emrE, eptA, evgS, gadW, gadX, H-NS, kdpE, mdf(A), mdtB, mdtC, mdtE, mdtF, mdtG, mdtM, msbA, pmrF, strA, ugd, yojI* | IS5 |
| CS_20 | Calf | A | A | 1122 | O154 | H48 | O154:H48 | *aslA, cfaB, cfaC, cfaD-E, cia, csgA-B, ecpA-E, ehaB, elfA-G, entA, entB, entC, espL1, espY, fdeC, fepA-C, fepD, fepG, fes, fimA, fimB-I, hcpA-C, hlyE, ibeB, ibeC, ompA, ompT, agn43* | *acrA, acrE, acrF, acrS, aph(3'')-Ib, baeS, bla-AmpC2, bla-AmpC1, bla-ampH, bla-TEM-1, bla-TEM-15, catA1, CRP, dfrA14, emrK, emrR, emrY, eptA, evgS, gadX, kdpE, mdf(A), mdtC, mdtE, mdtF, mdtG, mdtM, mdtN, mdtO, mdtP, mph(A), mph(B), msbA, oqxB, strB, sul2, tet(A), tet(B), yojI* | IS5 |
| CS_21 | Calf | A | A | 10 | O101 | H48 | O101:H48 | *aslA, cfaA, cfaB, cfaC, cia, csgA-B, ecpA-E, ehaB, elfA-G, entA, entB, entC, espL1, espY, fepA-C, fepD, fepG, fes, fimA, fimB-I, hcpA-C, hlyE, ibeB, ibeC, ompA, ompT, tia, afaA, agn43, fyuA, irp2, sitA-C, irpirp1, paa* | *aac(3)-IIa, acrA, acrB, aph(3'')-Ib, aph(6)-Id, bla-AmpC1, bla-ampH, bla-TEM-1, cpxA, dfrA1, dfrA17, emrE, emrK, eptA, evgS, fosA7, gadW, gadX, H-NS, mdtA, mdtB, mdtE, mdtH, mdtM, mdtN, mdtO, mdtP, mph(B), msbA, pmrF, qnrS1, sul2, sul1, tolC, ugd, yojI* | *IS5* |

| **Isolate** | **Sample** | **Clade** | **Phylogroup** | **STs** | **O-type** | **H-type** | **Serotypes** | **Virulence Gene(s) - VGs** | **Antibiotic resistance gene(s) - ARGs** | **OAGC - IS** |
| --- | --- | --- | --- | --- | --- | --- | --- | --- | --- | --- |
| CS_22 | Calf | B | B1 | 3576 | O16 | H48 | O16:H48 | *aslA, cfaA, cfaB, cfaC, csgA-B, ecpA-E, ehaB, elfA-G, entA, entB, entC, espL1, espY, fdeC, fepA-C, fepD, fepG, fes, fimA, fimB-I, hcpA-C, hlyE, ibeB, ibeC, ompA, ompT, agn43* | *acrD, acrE, aph(3'')-Ib, aph(6)-Id, baeS, bla-AmpC2, bla-AmpC1, bla-ampH, bla-PBP, cpxA, CRP, dfrA7, emrA, emrB, emrE, emrR, emrY, eptA, evgS, fosA7, gadW, gadX, H-NS, marA, mdf(A), mdtA, mdtB, mdtC, mdtH, mdtN, mdtO, mdtP, msbA, pmrF, qnrB6, qnrB17, ramA, strA, sul2, sul1, tet(A), tet(B), tetR, tolC, ugd* | *IS5* |
| CS_23 | Calf | C | E | 69 | O15 | H18 | O15:H18 | *aslA, cfaB, cfaC, cfaD-E, csgA-B, ecpA-E, elfA-G, entA, entB, entC, espL1, espY, fdeC, fepA-C, fepD, fepG, fes, fimA, fimB-I, hcpA-C, hlyA, hlyB, hlyC, hlyD, hlyE, ibeB, ibeC, iha, ompA, ompT, tia, fyuA, irp2, sitA-C, chuA, chuS, chuT, chuU, chuV, chuW, chuX, chuY, irpirp1, kpsM, papA, papB, papI, shuA, shuT, iroN, papC, iroB, iroC, iroD, iroE, ireA, papD, papE, papF, papG, papH, papJ, papK* | *aac(3)-IIa, acrS, aph(3'')-Ib, aph(6)-Id, bacA, baeR, baeS, bla-AmpC2, bla-ampH, bla-CTX-M-3, bla-TEM-1, cpxA, CRP, emrE, emrY, eptA, gadW, kdpE, marA, mdtB, mdtC, mdtE, mdtF, mdtG, mdtH, mdtM, mdtN, mdtO, mph(B), oqxA, strB, sul2, sul1, tet(B), tetR, tolC, yojI* | *-* |
| CS_24 | Calf | A | A | 1703 | O15 | H11 | O15:H11 | *aslA, csgA-B, ecpA-E, ehaB, elfA-G, entA, entB, entC, espL1, espY, fdeC, fepA-C, fepD, fepG, fes, fimA, fimB-I, hcpA-C, hlyE, ibeB, ibeC, ompA, ompT, aatA, agn43, sat, aap/aspU, aggR, astA, SefB, sepA, vat* | *aac(6')-Ib, acrA, acrB, acrD, acrF, acrS, aph(3'')-Ib, aph(6)-Id, arr-3, bacA, baeR, baeS, bla-AmpC1, bla-ampH, bla-CTX-M-3, bla-PBP, bla-TEM-15, catI, dfrA27, dfrA8, dfrA17, emrA, emrB, emrR, emrY, eptA, evgA, evgS, fosA, gadW, H-NS, kdpE, mdf(A), mdtB, mdtE, mdtF, mdtG, mdtN, mph(A), mph(B), strA, sul2, sul1, tet(A), tetR, tolC* | *IS5* |

| **Isolate** | **Sample** | **Clade** | **Phylogroup** | **STs** | **O-type** | **H-type** | **Serotypes** | **Virulence Gene(s) - VGs** | **Antibiotic resistance gene(s) - ARGs** | **OAGC - IS** |
| --- | --- | --- | --- | --- | --- | --- | --- | --- | --- | --- |
| CS_25 | Calf | A | A | 10 | O16 | H48 | O16:H48 | *aslA, cfaB, cfaC, cfaD-E, csgA-B, ecpA-E, ehaB, elfA-G, entA, entB, entC, espL1, espY, fdeC, fepA-C, fepD, fepG, fes, fimA, fimB-I, hcpA-C, hlyE, ibeB, ibeC, ompA, ompT, agn43* | *acrB, acrD, acrE, acrF, acrS, ant(3'')-Ia, aph(6)-Id, baeR, bla-AmpC2, bla-AmpC1, bla-PBP, bla-TEM-1, bla-TEM-15, bla-TEM-1B, cpxA, dfrA7, dfrA17, emrA, emrB, emrE, emrK, emrR, emrY, eptA, evgA, evgS, gadX, H-NS, kdpE, marA, mdf(A), mdtA, mdtC, mdtF, mdtG, mdtM, mdtN, mph(B), sul2, sul1, tet(A), tet(B), tetR, tolC, yojI* | *IS5* |
| CS_26 | Calf | A | A | 10 | O16 | H12 | O16:H12 | *aslA, cfaB, cfaC, csgA-B, ecpA-E, ehaB, elfA-G, entA, entB, entC, espL1, espY, fdeC, fepA-C, fepD, fepG, fes, fimA, fimB-I, hcpA-C, hlyE, ibeB, ibeC, ompA, ompT, aatA, agn43, fyuA, irp2, sitA-C, irpirp1* | *acrA, acrD, acrE, acrF, acrS, aph(3'')-Ib, bacA, baeR, bla-ampH, bla-CTX-M-3, bla-PBP, bla-SHV-145, bla-TEM-1, bla-TEM-15, bla-TEM-1B, cpxA, dfrA7, dfrA14, emrA, emrK, emrR, emrY, eptA, evgA, evgS, gadX, mdf(A), mdtB, mdtE, mdtG, mdtH, mdtM, mdtO, mph(B), oqxB, pmrF, tet(A), tet(B), tolC, yojI* | *-* |
| CS_27 | Calf | A | A | 398 | O16 | H48 | O16:H48 | *aslA, cfaB, cfaC, cfaD-E, csgA-B, ecpA-E, ehaB, elfA-G, entA, entB, entC, espL1, espY, fdeC, fepA-C, fepD, fepG, fes, fimA, fimB-I, hcpA-C, hlyE, ibeB, ibeC, ompA, ompT, agn43* | *aac(3)-IIe, acrA, acrE, acrF, acrS, aph(3'')-Ib, aph(6)-Id, bla-AmpC2, bla-AmpC1, bla-ampH, bla-PBP, bla-TEM-1, bla-TEM-15, bla-TEM-1B, CRP, dfrA7, emrB, emrE, emrY, gadX, kdpE, marA, mdf(A), mdtA, mdtB, mdtC, mdtE, mdtF, mdtM, mdtO, mdtP, msbA, sul1, tet(A), tetR, tolC, yojI* | *IS5* |

| **Isolate** | **Sample** | **Clade** | **Phylogroup** | **STs** | **O-type** | **H-type** | **Serotypes** | **Virulence Gene(s) - VGs** | **Antibiotic resistance gene(s) - ARGs** | **OAGC - IS** |
| --- | --- | --- | --- | --- | --- | --- | --- | --- | --- | --- |
| CS_28 | Calf | A | A | 10 | O16 | H48 | O16:H48 | *aslA, csgA-B, ecpA-E, elfA-G, entA, entB, entC, espL1, espY, fepA-C, fepD, fepG, fes, fimA, fimB-I, hcpA-C, hlyA, hlyB, hlyD, hlyE, ibeB, ibeC, ompA, ompT, stx2, agn43, stx1, eae, nleB, nleE, nleF, nleH* | *acrA, acrB, acrF, acrS, aph(3'')-Ib, aph(6)-Id, bacA, baeR, baeS, bla-AmpC1, bla-ampH, bla-PBP, bla-TEM-1, bla-TEM-15, bla-TEM-1B, catA1, CRP, dfrA7, dhfr7, emrK, emrR, emrY, evgA, gadW, H-NS, kdpE, mdtB, mdtE, mdtH, mdtM, mdtN, mdtO, mdtP, mph(B), pmrF, strA, strB, tet(A), tet(B), tetR, tolC* | *IS5* |
| CS_29 | Calf | B | B1 | 847 | O16 | H48 | O16:H48 | *aslA, cfaA, cfaB, cfaC, cfaD-E, csgA-B, ecpA-E, ehaA, elfA-G, entA, entB, entC, espL1, espY, fdeC, fepA-C, fepD, fepG, fes, fimA, fimB-I, hcpA-C, hlyE, ibeB, ibeC, ompA, ompT, agn43* | *acrA, acrD, aph(6)-Id, bacA, baeR, bla-AmpC1, bla-ampH, bla-PBP, bla-TEM-1, CRP, dfrA7, emrA, emrB, emrK, emrR, eptA, evgA, evgS, gadX, H-NS, kdpE, marA, mdf(A), mdtA, mdtC, mdtE, mdtF, mdtG, mdtH, mdtO, mdtP, pmrF, sul2, sul1, tet(A), tet(B), tetR, tolC, yojI* | *IS5* |
| CS_30 | Calf | B | B1 | 155 | O16 | H48 | O16:H48 | *aslA, cdiAB, cfaA, cfaB, cfaC, csgA-B, ecpA-E, ehaA, elfA-G, entA, entB, entC, espL1, espY, fdeC, fepA-C, fepD, fepG, fes, fimA, fimB-I, hcpA-C, hlyE, ibeB, ibeC, ompA, ompT, tia, afaA, afaC, agn43, iucC, aafC, afaB, iucD, papX, iroN, iroB, FF17d7d, tibA, aafB, afaF* | *acrA, acrB, acrD, acrE, acrF, aph(3'')-Ib, aph(6)-Id, bacA, baeS, bla-AmpC1, bla-ampH, bla-PBP, bla-TEM-1, CRP, dfrA7, dfrA8, dfrA1, dfrA14, dhfr7, emrE, emrY, eptA, evgA, gadX, H-NS, marA, mdf(A), mdtA, mdtB, mdtC, mdtF, mdtG, mdtH, mdtM, mph(B), msbA, strA, sul2, sul1, tet(A), tet(B), tetR, yojI* | *IS5* |

| **Isolate** | **Sample** | **Clade** | **Phylogroup** | **STs** | **O-type** | **H-type** | **Serotypes** | **Virulence Gene(s) - VGs** | **Antibiotic resistance gene(s) - ARGs** | **OAGC - IS** |
| --- | --- | --- | --- | --- | --- | --- | --- | --- | --- | --- |
| CS_31 | Calf | A | A | 1312 | O16 | H48 | O16:H48 | *aslA, csgA-B, ecpA-E, ehaB, elfA-G, entA, entB, entC, espL1, espY, fdeC, fepA-C, fepD, fepG, fes, fimA, fimB-I, hcpA-C, hlyE, ibeB, ibeC, ompA, ompT, aatA, agn43, pic, kpsD, kpsM, aai-A/M, aap/aspU, aggR, astA* | *aac(3)-IIe, acrB, acrD, acrE, acrF, acrS, aph(3'')-Ib, aph(6)-Id, bacA, baeR, bla-AmpC1, bla-PBP, bla-TEM-1, dfrA7, dfrA8, emrE, emrK, eptA, gadW, gadX, H-NS, mdf(A), mdtE, mdtF, mdtG, mdtH, mdtM, mdtN, mdtO, msbA, strA, strB, sul2, sul1, tet(A), tet(B), tetR, tolC, ugd* | *IS5* |
| ES_01 | Enviroment | A | A | 227 | O16 | H48 | O16:H48 | *aslA, cia, csgA-B, ecpA-E, ehaB, elfA-G, entA, entB, entC, espL1, espY, fdeC, fepA-C, fepD, fepG, fes, fimA, fimB-I, hcpA-C, hlyE, ibeB, ibeC, ompA, ompT, agn43, iucC, iutA, sitA-C, iucA, iucB, iucD, aap/aspU, aggR, iroN, iroB, iroC, iroD, iroE* | *acrB, acrD, acrE, acrS, aph(3'')-Ib, baeR, baeS, bla-AmpC1, bla-CTX-M-15, bla-PBP, bla-TEM-1, bla-TEM-15, catA1, cpxA, emrE, emrR, emrY, eptA, evgA, kdpE, marA, mdf(A), mdtB, mdtC, mdtE, mdtF, mdtG, mdtH, mdtM, mdtN, mdtO, mph(B), msbA, pmrF, strA, strB, tet(A), ugd, yojI* | *-* |
| ES_02 | Enviroment | A | A | 227 | O16 | H48 | O16:H48 | *aslA, cfaA, cfaB, cfaC, cia, csgA-B, ecpA-E, ehaB, elfA-G, entA, entB, entC, espL1, espY, fdeC, fepA-C, fepD, fepG, fes, fimA, fimB-I, hcpA-C, hlyE, ibeB, ibeC, ompA, ompT, agn43, iucC, iutA, sitA-C, iucA, iucB, iucD, iroN, iroB, iroC, iroD, iroE* | *acrA, acrB, acrF, aph(3'')-Ib, baeR, bla-AmpC2, bla-AmpC1, bla-ampH, bla-CTX-M-15, bla-TEM-1, bla-TEM-1B, catA1, catI, cpxA, CRP, emrE, emrY, eptA, evgA, gadW, H-NS, kdpE, marA, mdf(A), mdtA, mdtB, mdtC, mdtF, mdtG, mdtM, mdtN, mdtO, msbA, pmrF, sul2, tet(A), tolC, ugd, yojI* | *-* |

| **Isolate** | **Sample** | **Clade** | **Phylogroup** | **STs** | **O-type** | **H-type** | **Serotypes** | **Virulence Gene(s) - VGs** | **Antibiotic resistance gene(s) - ARGs** | **OAGC - IS** |
| --- | --- | --- | --- | --- | --- | --- | --- | --- | --- | --- |
| ES_03 | Enviroment | B | B1 | 58 | O16 | H48 | O16:H48 | *aslA, cfaA, cfaB, cfaC, csgA-B, ecpA-E, ehaA, elfA-G, entA, entB, entC, espL1, espY, fdeC, fepA-C, fepD, fepG, fes, fimA, fimB-I, hcpA-C, hlyE, ibeB, ibeC, ompA, ompT, agn43, iucC, iutA, sitA-C, iucA, iucB, iucD, iroN, iroB, iroC, iroD, iroE, vat, tsh* | *aac(6')-Ib, aadA16, acrA, acrB, acrD, acrF, acrS, aph(3'')-Ib, bacA, baeR, bla-AmpC2, bla-AmpC1, bla-ampH, bla-CTX-M-3, bla-PBP, bla-TEM-1, bla-TEM-15, bla-TEM-1B, catA1, CRP, dfrA14, emrE, emrY, eptA, evgA, evgS, gadW, gadX, marA, mdf(A), mdtB, mdtE, mdtH, mdtM, mdtP, mph(B), strA, strB, sul2, yojI* | *IS5* |
| ES_04 | Enviroment | B | B1 | 56 | O16 | H48 | O16:H48 | *aslA, cfaA, cfaB, cfaC, cfaD-E, csgA-B, ecpA-E, ehaA, ehaB, elfA-G, entA, entB, entC, espL1, espY, fdeC, fepA-C, fepD, fepG, fes, fimA, fimB-I, hcpA-C, hlyE, ibeB, ibeC, ompA, ompT, tia, agn43, fyuA, irp2, irpirp1, papX, FF17d7d, tibA, afaF* | *aadA1, acrB, acrD, acrE, aph(3'')-Ib, bacA, bla-AmpC1, bla-ampH, bla-PBP, catA1, cpxA, CRP, emrA, emrB, emrK, emrR, emrY, eptA, evgS, H-NS, kdpE, marA, mdf(A), mdtA, mdtF, mdtN, mdtO, mdtP, msbA, pmrF, tet(A), tet(B), tetR, tolC, yojI* | *IS5* |
| ES_05 | Enviroment | B | B1 | 1049 | O16 | H10 | O16:H10 | *aslA, cfaA, cfaB, cfaC, csgA-B, ecpA-E, ehaA, elfA-G, entA, entB, entC, espL1, espY, fdeC, fepA-C, fepD, fepG, fes, fimA, fimB-I, hcpA-C, hlyE, ibeB, ibeC, ompA, ompT, aatA, agn43, sitA-C* | *acrA, acrB, acrE, acrF, acrS, aph(3'')-Ib, aph(6)-Id, baeS, bla-AmpC2, bla-AmpC1, bla-ampH, bla-TEM-15, catA1, cpxA, CRP, dfrA7, dfrA17, emrA, emrB, emrE, emrK, emrY, evgS, gadW, gadX, kdpE, marA, mdf(A), mdtA, mdtC, mdtE, mdtF, mdtM, mdtO, mph(A), msbA, strA, sul2, tet(A), ugd* | *IS5* |

| **Isolate** | **Sample** | **Clade** | **Phylogroup** | **STs** | **O-type** | **H-type** | **Serotypes** | **Virulence Gene(s) - VGs** | **Antibiotic resistance gene(s) - ARGs** | **OAGC - IS** |
| --- | --- | --- | --- | --- | --- | --- | --- | --- | --- | --- |
| ES_06 | Enviroment | A | A | 450 | O16 | H16 | O16:H16 | *aslA, cfaA, cfaB, cfaC, csgA-B, ecpA-E, elfA-G, entA, entB, entC, espL1, espY, fdeC, fepA-C, fepD, fepG, fes, fimA, fimB-I, hcpA-C, hlyE, ibeB, ibeC, iha, ompA, ompT, agn43, iucC, iutA, iucA, iucB, iucD, kpsD, kpsM, papA, papB, papI, papX, sat, papE, papF* | *aadA5, acrB, acrD, acrF, acrS, aph(6)-Id, bacA, baeR, baeS, bla-AmpC1, bla-CTX-M-15, bla-TEM-1, bla-TEM-1B, cpxA, dfrA1, dfrA14, dhfr7, emrB, emrE, emrK, evgA, evgS, H-NS, kdpE, marA, mdf(A), mdtA, mdtB, mdtC, mdtE, mdtM, mdtN, mdtO, msbA, strA, sul2, sul1, tet(A), tet(B), ugd, yojI* | *IS5* |
| ES_07 | Enviroment | A | A | 409 | O75 | H55 | O75:H55 | *aslA, cfaD-E, csgA-B, ecpA-E, ehaB, elfA-G, entA, entB, entC, espL1, espY, fepA-C, fepD, fepG, fes, fimA, fimB-I, hcpA-C, hlyE, ibeB, ibeC, ompA, ompT, agn43, sitA-C* | *aadA5, acrA, acrB, acrE, acrF, aph(6)-Id, baeR, bla-AmpC1, bla-ampH, bla-PBP, bla-TEM-1, cpxA, CRP, emrB, emrE, emrK, emrY, eptA, evgA, gadW, H-NS, marA, mdf(A), mdtA, mdtB, mdtC, mdtE, mdtF, mdtG, mdtH, mdtM, mdtP, msbA, pmrF, strB, sul2, sul1, tet(A), yojI* | *IS5* |
| ES_08 | Enviroment | A | A | 176 | O16 | H48 | O16:H48 | *aslA, cfaA, cfaB, cfaC, cfaD-E, csgA-B, ecpA-E, ehaB, elfA-G, entA, entB, entC, espL1, espY, fdeC, fepA-C, fepD, fepG, fes, fimA, fimB-I, hcpA-C, hlyE, ibeB, ibeC, ompA, ompT, agn43* | *aac(3)-IIe, aadA16, acrA, acrB, acrD, acrF, acrS, aph(3'')-Ib, aph(6)-Id, bla-AmpC2, bla-AmpC1, bla-ampH, bla-OXA, bla-PBP, bla-SHV-145, bla-TEM-1, bla-TEM-15, bla-TEM-1B, catI, dfrA7, emrA, emrE, eptA, evgA, gadW, kdpE, marA, mdtB, mdtC, mdtE, mdtF, mph(B), sul2, tet(A), tetR, tolC, ugd* | *-* |

| **Isolate** | **Sample** | **Clade** | **Phylogroup** | **STs** | **O-type** | **H-type** | **Serotypes** | **Virulence Gene(s) - VGs** | **Antibiotic resistance gene(s) - ARGs** | **OAGC - IS** |
| --- | --- | --- | --- | --- | --- | --- | --- | --- | --- | --- |
| ES_09 | Enviroment | B | B1 | 906 | O16 | H48 | O16:H48 | *aslA, cfaA, cfaB, cfaC, csgA-B, ecpA-E, ehaA, ehaB, elfA-G, entA, entB, entC, espL1, espY, fdeC, fepA-C, fepD, fepG, fes, fimA, fimB-I, hcpA-C, hlyE, ibeB, ibeC, ompA, ompT, papA, papC, tibA, papD, papH* | *acrB, acrD, acrE, aph(6)-Id, bacA, baeR, baeS, bla-AmpC1, bla-ampH, bla-PBP, bla-TEM-1C, CRP, dfrA7, emrB, emrE, emrK, emrY, evgA, gadW, kdpE, mdf(A), mdtA, mdtC, mdtF, mdtG, mdtH, mdtN, msbA, pmrF, strA, sul2, sul1, tet(A), tet(B), tolC, ugd* | *IS5* |
| ES_10 | Enviroment | B | B1 | 156 | O115 | H48 | O115:H48 | *aslA, cdiAB, cfaA, cfaB, cfaC, cfaD-E, csgA-B, ecpA-E, ehaB, elfA-G, entA, entB, entC, espL1, espY, fdeC, fepA-C, fepD, fepG, fes, fimA, fimB-I, hcpA-C, hlyE, ibeB, ibeC, ompA, ompT, afaC, fyuA, irp2, afaB, cah, irpirp1, aai-A/M* | *aadA5, acrA, acrB, acrD, acrE, acrS, aph(3'')-Ib, bacA, baeR, baeS, bla-AmpC2, bla-AmpC1, bla-ampH, bla-TEM-1, bla-TEM-135, cpxA, dfrA7, dfrA14, emrE, emrK, emrR, emrY, eptA, evgA, gadW, gadX, mdf(A), mdtA, mdtB, mdtC, mdtE, mdtG, mdtH, mdtM, mdtN, mdtP, mph(B), pmrF, sat-2, strA, sul2, sul1, tet(B), ugd, yojI* | *IS5* |
| ES_11 | Enviroment | B | B1 | 3576 | O16 | H48 | O16:H48 | *aslA, cfaA, cfaB, cfaC, csgA-B, ecpA-E, ehaB, elfA-G, entA, entB, entC, espL1, espY, fdeC, fepA-C, fepD, fepG, fes, fimA, fimB-I, hcpA-C, hlyE, ibeB, ibeC, ompA, ompT, agn43, cah* | *acrB, acrD, acrS, aph(3'')-Ib, aph(6)-Id, baeS, bla-AmpC2, bla-AmpC1, bla-ampH, bla-OXA, bla-TEM-1, cpxA, emrA, emrB, emrE, emrY, eptA, gadW, gadX, H-NS, kdpE, marA, mdf(A), mdtA, mdtB, mdtC, mdtE, mdtF, mdtH, mdtM, mdtN, mdtO, mph(A), mph(B), msbA, pmrF, sul2, sul1, tet(A), tet(B), tolC, ugd, yojI* | *IS5* |

|  |  |  |  |  |  |  |  |  |  |  |
| --- | --- | --- | --- | --- | --- | --- | --- | --- | --- | --- |
| ES_12 | Enviroment | A | A | 1112 | O154 | H10 | O154:H10 | *aslA, cfaB, cfaC, cfaD-E, cia, csgA-B, ecpA-E, ehaB, elfA-G, entA, entB, entC, espL1, espY, fdeC, fepA-C, fepD, fepG, fes, fimA, fimB-I, hcpA-C, hlyC, hlyE, ibeB, ibeC, ompA, ompT, agn43* | *acrA, acrF, acrS, aph(3'')-Ib, aph(6)-Id, bacA, baeS, bla-AmpC1, bla-ampH, bla-PBP, bla-TEM-15, bla-TEM-1B, cpxA, CRP, dfrA1, emrB, emrK, eptA, evgA, gadX, H-NS, mdf(A), mdtB, mdtC, mdtE, mdtF, mdtG, mdtH, mdtM, mph(B), msbA, oqxB2, strB, sul2, tet(A), tolC, yojI* | IS5 |
| ES_13 | Enviroment | A | A | 480 | O156 | H48 | O156:H48 | *aslA, cfaA, cfaB, cfaC, cfaD-E, csgA-B, ecpA-E, ehaB, elfA-G, entA, entB, entC, espL1, espY, fepA-C, fepD, fepG, fes, fimA, fimB-I, hcpA-C, hlyE, ibeB, ibeC, ompA, ompT, agn43* | *acrB, acrE, acrF, acrS, aph(3'')-Ib, aph(6)-Id, arr-3, bacA, baeR, baeS, bla-AmpC2, bla-AmpC1, bla-ampH, bla-PBP, bla-TEM-1, bla-TEM-1B, catA1, dfrA7, dfrA14, emrA, emrE, emrK, emrR, emrY, eptA, gadX, mdtB, mdtC, mdtE, mdtF, mdtM, mdtN, mph(B), msbA, sul2, sul1, tet(A), tet(B), tolC, ugd* | IS5 |
| ES_14 | Enviroment | B | B1 | 155 | O16 | H48 | O16:H48 | *aslA, csgA-B, ecpA-E, ehaA, ehaB, elfA-G, entA, entB, entC, espL1, espY, fdeC, fepA-C, fepD, fepG, fes, fimA, fimB-I, hcpA-C, hlyE, ibeB, ibeC, ompA, ompT, aatA, sat, aap/aspU, aggR, astA, fagC, SefB, sepA, vat* | *aac(3)-IIa, acrA, acrD, acrE, acrF, acrS, aph(3'')-Ib, aph(6)-Id, baeR, baeS, bla-AmpC1, bla-ampH, bla-CTX-M-15, bla-PBP, bla-TEM-235, bla-TEM-1, CRP, dfrA14, dfrA15, dfrA17, emrE, emrK, emrR, eptA, evgS, H-NS, kdpE, marA, mdf(A), mdtA, mdtB, mdtC, mdtF, mdtN, mdtO, mdtP, mph(A), mph(B), sat-2, sul2, sul1, tet(A), tet(B), ugd* | IS5 |

| **Isolate** | **Sample** | **Clade** | **Phylogroup** | **STs** | **O-type** | **H-type** | **Serotypes** | **Virulence Gene(s) - VGs** | **Antibiotic resistance gene(s) - ARGs** | **OAGC - IS** |
| --- | --- | --- | --- | --- | --- | --- | --- | --- | --- | --- |
| ES_15 | Enviroment | B | B1 | 3576 | O16 | H48 | O16:H48 | *aslA, cfaA, cfaB, cfaC, csgA-B, ecpA-E, ehaB, elfA-G, entA, entB, entC, espL1, espY, fdeC, fepA-C, fepD, fepG, fes, fimA, fimB-I, hcpA-C, hlyE, ibeB, ibeC, ompA, ompT, agn43* | *acrA, acrB, acrE, acrF, aph(6)-Id, bacA, baeR, bla-AmpC2, bla-AmpC1, bla-ampH, bla-OXA, bla-PBP, bla-TEM-12, bla-TEM-1D, cpxA, CRP, emrB, emrR, emrY, eptA, gadW, gadX, H-NS, kdpE, marA, mdf(A), mdtB, mdtE, mdtF, mdtH, mdtM, mdtN, mdtO, mdtP, mph(B), msbA, strA, strB, sul2, sul1, tet(A), tolC, yojI* | *IS5* |
| ES_16 | Enviroment | B | B1 | 155 | O16 | H21 | O16:H21 | *aslA, cdiAB, cfaA, cfaB, cfaC, cfaD-E, csgA-B, ecpA-E, ehaA, ehaB, elfA-G, entA, entB, entC, espL1, espY, fdeC, fepA-C, fepD, fepG, fes, fimA, fimB-I, hcpA-C, hlyE, ibeB, ibeC, ompA, ompT, tia, agn43, papX, iroN* | *acrA, acrB, acrD, acrE, acrF, acrS, aph(3'')-Ib, aph(6)-Id, baeR, baeS, bla-AmpC1, bla-ampH, bla-CTX-M-3, bla-TEM-1, catA1, cpxA, dfrA7, dfrA14, dfrA15, emrA, emrE, emrR, emrY, eptA, evgA, evgS, gadW, gadX, marA, mdf(A), mdtB, mdtE, mdtF, mdtN, mdtO, mdtP, mph(B), oqxA11, sul2, sul1, tet(A), tet(B), tolC, yojI* | *IS5* |
| ES_17 | Enviroment | B | B1 | 101 | O16 | H48 | O16:H48 | *aslA, cdiAB, csgA-B, ecpA-E, ehaA, ehaB, elfA-G, entA, entB, entC, espL1, espY, fdeC, fepA-C, fepD, fepG, fes, fimA, fimB-I, hcpA-C, hlyA, hlyC, hlyE, ibeB, ibeC, iha, ompA, ompT, tia, afaA, afaC, afaD, iucC, iutA, aafC, afaB, afaE, iucA, iucB, iucD, papX, cdtA, cdtB, cdtC, cnfcnf1-2-2, tibA, stx, stx1, afaF* | *acrB, aph(6)-Id, bacA, baeR, bla-AmpC1, bla-ampH, bla-PBP, bla-TEM-15, bla-TEM-1B, cpxA, CRP, dfrA7, dfrA8, dfrA14, emrA, emrB, emrE, emrR, emrY, eptA, evgA, gadW, gadX, H-NS, mdf(A), mdtB, mdtC, mdtE, mdtF, mdtM, mdtN, mdtP, msbA, pmrF, sat-2, sul2, sul1, tet(A), tet(B), tolC, ugd, yojI* | *IS5* |

| **Isolate** | **Sample** | **Clade** | **Phylogroup** | **STs** | **O-type** | **H-type** | **Serotypes** | **Virulence Gene(s) - VGs** | **Antibiotic resistance gene(s) - ARGs** | **OAGC - IS** |
| --- | --- | --- | --- | --- | --- | --- | --- | --- | --- | --- |
| ES_18 | Enviroment | B | A | 10 | O107 | H48 | O107:H48 | *aslA, csgA-B, ecpA-E, ehaB, elfA-G, entA, entB, entC, espL1, espY, fdeC, fepA-C, fepD, fepG, fes, fimA, fimB-I, hcpA-C, hlyE, ibeB, ibeC, iha, ompA, ompT, iucC, iutA, pic, cah, iucA, iucB, iucD, aai-A/M, astA* | *aac(3)-IIa, aac(3)-IIe, acrA, acrF, acrS, ant(3'')-Ia, aph(3'')-Ib, aph(6)-Id, baeR, baeS, bla-AmpC1, bla-ampH, bla-PBP, bla-TEM-15, cpxA, CRP, dfrA15, dhfr7, emrK, emrR, evgA, evgS, gadX, H-NS, marA, mdf(A), mdtB, mdtC, mdtE, mdtM, mdtP, mph(A), pmrF, sul2, sul1, tet(A), tetR, tolC, yojI* | *IS5* |
| ES_19 | Enviroment | E | A | 1703 | O16 | H48 | O16:H48 | *aslA, csgA-B, ecpA-E, elfA-G, entA, entB, entC, espL1, espY, fdeC, fepA-C, fepD, fepG, fes, fimA, fimB-I, hcpA-C, hlyE, ibeB, ibeC, iha, ompA, ompT, aatA, agn43, fyuA, irp2, iucC, iutA, pic, sitA-C, aafC, chuS, chuU, irpirp1, senB, aai-A/M, fagC, iroN, iroE, stx, stx1, aafB, aafA, aafD, pet, shuV, ybtA* | *aac(3)-IIe, acrA, acrB, acrE, acrS, aph(3'')-Ib, aph(6)-Id, bacA, baeR, bla-AmpC2, bla-AmpC1, bla-ampH, bla-PBP, bla-TEM-1, bla-TEM-15, dfrA17, emrA, emrB, emrE, emrK, emrY, evgA, evgS, gadW, gadX, H-NS, kdpE, mdf(A), mdtA, mdtB, mdtC, mdtF, mdtG, mdtH, mdtM, mdtN, mdtO, mdtP, mph(B), pmrF, strA, strB, sul2, sul1, tet(A), tet(B), yojI* | *IS5* |
| ES_20 | Enviroment | C | B | 201 | O136 | H19 | O136:H19 | *aslA, cfaA, cfaB, cfaC, cia, csgA-B, ecpA-E, ehaA, ehaB, elfA-G, entA, entB, entC, espL1, espY, fdeC, fepA-C, fepD, fepG, fes, fimA, fimB-I, hcpA-C, hlyA, hlyB, hlyC, hlyD, hlyE, ibeB, ibeC, iha, ompA, ompT, stx2, agn43, stx, stx1* | *acrA, acrD, acrE, acrF, aph(6)-Id, bacA, bla-AmpC2, bla-AmpC1, bla-CTX-M-3, bla-PBP, bla-TEM-1, catII, cpxA, CRP, dfrA1, emrE, emrY, evgA, evgS, gadW, gadX, kdpE, marA, mdf(A), mdtA, mdtC, mdtE, mdtF, mdtG, mdtN, mdtP, msbA, pmrF, strA, sul2, tet(A), tolC, yojI* | *IS5* |

| **Isolate** | **Sample** | **Clade** | **Phylogroup** | **STs** | **O-type** | **H-type** | **Serotypes** | **Virulence Gene(s) - VGs** | **Antibiotic resistance gene(s) - ARGs** | **OAGC - IS** |
| --- | --- | --- | --- | --- | --- | --- | --- | --- | --- | --- |
| ES_21 | Enviroment | C | B | 869 | O16 | H48 | O16:H48 | *aslA, cfaA, cfaB, cfaC, cfaD-E, csgA-B, ecpA-E, ehaA, ehaB, elfA-G, entA, entB, entC, espL1, espY, fdeC, fepA-C, fepD, fepG, fes, fimA, fimB-I, hcpA-C, hlyE, ibeB, ibeC, ompA, ompT, agn43, eltA* | *aac(3)-IIe, acrA, acrD, acrF, acrS, aph(3'')-Ib, aph(6)-Id, baeR, bla-AmpC2, bla-AmpC1, bla-ampH, bla-CTX-M-3, bla-PBP, bla-TEM-1, cpxA, dfrA7, emrA, emrB, emrK, emrR, emrY, eptA, fosA6, gadW, H-NS, kdpE, marA, mdf(A), mdtB, mdtE, mdtF, mdtG, mdtM, mdtN, mdtO, pmrF, strA, sul2, sul1, tet(A), tet(B), tolC* | *IS5* |
| ES_22 | Enviroment | B | Unknown | 300 | O156 | H48 | O156:H48 | *aslA, csgA-B, ecpA-E, ehaA, ehaB, elfA-G, entA, entB, entC, espL1, espY, fdeC, fepA-C, fepD, fepG, fes, fimA, fimB-I, hcpA-C, hlyA, hlyB, hlyC, hlyD, hlyE, ibeB, ibeC, ompA, ompT, agn43, sepA, paa, eae, nleB, nleF, nleH, cesAB, cesD, east1, escV, espA, espF, map, nleAA, nleC, nleG, tir* | *acrA, acrB, acrD, acrE, acrS, aph(3'')-Ib, aph(6)-Id, bla-AmpC2, bla-ampH, bla-TEM-235, bla-TEM-15, dfrA7, emrE, emrK, emrY, eptA, evgA, evgS, gadW, gadX, H-NS, mdf(A), mdtB, mdtE, mdtF, mdtM, mdtN, mdtP, mph(B), sul1, tet(A), tetR, tolC, ugd, yojI* | *IS5* |
| HS_01 | Human | C | E | 349 | O16 | H15 | O16:H15 | *aslA, csgA-B, ecpA-E, ehaB, elfA-G, entA, entB, entC, espL1, espY, fdeC, fepA-C, fepD, fepG, fes, fimA, fimB-I, hcpA-C, hlyE, ibeB, ibeC, ompA, ompT, tia, aatA, afaC, afaD, agn43, chuA, chuS, chuT, chuU, chuV, chuW, chuX, chuY, kpsD, kpsM, shuA, shuT, iroN, iroD, iroE, agg3D, shuS* | *acrA, acrB, acrD, acrF, aph(3'')-Ib, aph(6)-Id, bacA, bla-AmpC1, bla-ampH, bla-CTX-M-15, bla-PBP, bla-TEM-1B, cpxA, CRP, dfrA7, dhfr7, emrA, emrB, emrE, eptA, evgA, gadW, gadX, H-NS, kdpE, mdf(A), mdtA, mdtE, mdtH, mdtM, mdtP, msbA, sul2, tet(A), tet(B), tet(D), tetR, ugd* |  |

| **Isolate** | **Sample** | **Clade** | **Phylogroup** | **STs** | **O-type** | **H-type** | **Serotypes** | **Virulence Gene(s) - VGs** | **Antibiotic resistance gene(s) - ARGs** | **OAGC - IS** |
| --- | --- | --- | --- | --- | --- | --- | --- | --- | --- | --- |
| HS_02 | Human | A | A | 656 | O16 | H10 | O16:H10 | *aslA, csgA-B, ecpA-E, elfA-G, entA, entB, entC, espL1, espY, fepA-C, fepD, fepG, fes, fimA, fimB-I, hcpA-C, hlyE, ibeB, ibeC, ompA, ompT, tia, afaA, afaC, afaD, agn43, fyuA, irp2, iucC, iutA, sitA-C, aafC, afaB, afaE, daaF, draD, irpirp1, iucA, iucB, iucD, papI, draA, draC* | *acrA, acrB, aph(3'')-Ib, aph(6)-Id, baeR, baeS, bla-AmpC2, bla-ampH, bla-CTX-M-15, bla-PBP, bla-TEM-1, bla-TEM-1B, CRP, dfrA14, dfrA17, dhfr7, emrK, emrR, emrY, eptA, evgA, evgS, H-NS, marA, mdf(A), mdtA, mdtB, mdtF, mdtM, mdtP, mph(B), msbA, pmrF, sul2, tet(A), tolC, yojI* | *IS5* |
| HS_03 | Human | A | A | 10 | O16 | H48 | O16:H48 | *aslA, cdiAB, csgA-B, ecpA-E, ehaB, elfA-G, entA, entB, entC, espL1, espY, fdeC, fepA-C, fepD, fepG, fes, fimA, fimB-I, hcpA-C, hlyE, ibeB, ibeC, iha, ompA, ompT, afaA, afaC, afaD, agn43, fyuA, irp2, iucC, iutA, sitA-C, afaB, chuA, irpirp1, iucA, iucB, iucD, kpsM, papA, sat, aai-A/M, aggR, astA, fagC, iroB, ybtA* | *aac(6')-Ib, acrA, acrD, acrE, acrF, acrS, aph(3'')-Ib, bacA, baeR, baeS, bla-AmpC1, bla-ampH, bla-CTX-M-15, bla-PBP, bla-TEM-1B, cpxA, dfrA7, dhfr7, evgA, evgS, kdpE, marA, mdf(A), mdtA, mdtB, mdtC, mdtE, mdtG, mdtH, mdtO, mdtP, mph(B), msbA, pmrF, sul1, tet(A), tetR, ugd, yojI* | *-* |
| HS_04 | Human | C | E | UNK | O16 | H48 | O16:H48 | *aslA, csgA-B, ecpA-E, ehaA, elfA-G, entA, entB, entC, espL1, espY, fdeC, fepA-C, fepD, fepG, fimA, fimB-I, hcpA-C, hlyA, hlyB, hlyC, hlyD, hlyE, ibeC, iha, ompA, ompT, tia, aatA, afaC, afaD, agn43, iucC, iutA, chuA, chuS, chuT, chuU, chuV, chuW, chuX, chuY, iucA, iucB, iucD, kpsD, kpsM, shuA, astA, shuT, iroN, iroD, iroE, sepA, paa, eae, nleB, nleE, nleF, nleH, cesAB, cesD, escV, espA, map, nleAA, nleC, nleG, tir, shuS, lifa/efalifa/efa1, katP* | *acrA, acrB, acrD, aph(3'')-Ib, aph(6)-Id, bacA, baeS, bla-AmpC2, bla-AmpC1, bla-ampH, bla-OXA, bla-PBP, bla-TEM-15, emrE, emrR, eptA, evgA, gadW, gadX, mdtE, mdtF, mdtN, mdtP, pmrF, strA, strB, sul2, sul1, tet(A), tet(D), tetR, ugd* | *IS5* |

| **Isolate** | **Sample** | **Clade** | **Phylogroup** | **STs** | **O-type** | **H-type** | **Serotypes** | **Virulence Gene(s) - VGs** | **Antibiotic resistance gene(s) - ARGs** | **OAGC - IS** |
| --- | --- | --- | --- | --- | --- | --- | --- | --- | --- | --- |
| HS_05 | Human | C | E | 349 | O16 | H16 | O16:H16 | *aslA, csgA-B, ecpA-E, ehaB, elfA-G, entA, entB, entC, espL1, espY, fdeC, fepA-C, fepD, fepG, fes, fimA, fimB-I, hcpA-C, hlyE, ibeB, ibeC, ompA, ompT, tia, aatA, afaC, afaD, agn43, chuA, chuS, chuT, chuU, chuV, chuW, chuX, chuY, kpsD, kpsM, shuA, shuT, iroN, iroD, iroE, agg3D, shuS* | *acrA, acrB, acrD, acrE, acrF, acrS, aph(3'')-Ib, aph(6)-Id, bacA, baeR, bla-CTX-M-15, bla-OXA, bla-PBP, bla-TEM-1, bla-TEM-1C, cpxA, CRP, dfrA7, emrA, emrB, emrE, emrR, emrY, eptA, gadW, gadX, H-NS, kdpE, mdtA, mdtB, mdtE, mdtG, mdtN, mdtO, mph(B), msbA, pmrF, sul2, tet(A), tet(B), tet(D), ugd, yojI* | *-* |
| HS_06 | Human | A | A | 1495 | O16 | H48 | O16:H48 | *aslA, cfaB, cfaC, cfaD-E, csgA-B, ecpA-E, ehaB, elfA-G, entA, entB, entC, espL1, espY, fdeC, fepA-C, fepD, fepG, fes, fimA, fimB-I, hcpA-C, hlyE, ibeB, ibeC, ompA, ompT, tia, agn43* | *acrE, acrF, acrS, aph(3'')-Ib, bacA, baeR, bla-AmpC2, bla-AmpC1, bla-ampH, bla-PBP, CRP, dfrA5, emrB, emrE, emrY, eptA, evgS, gadX, H-NS, mdf(A), mdtA, mdtB, mdtE, mdtF, mdtM, mdtN, mdtO, mdtP, mph(A), msbA, strA, sul2, tet(B), tolC, ugd* | *IS5* |
| HS_07 | Human | A | A | 10 | O11 | H48 | O11:H48 | *aslA, csgA-B, ecpA-E, ehaB, elfA-G, entA, entB, entC, espL1, espY, fdeC, fepA-C, fepD, fepG, fes, fimA, fimB-I, hcpA-C, hlyE, ibeB, ibeC, iha, ompA, ompT, agn43, fyuA, irp2, iucC, iutA, sitA-C, irpirp1, iucA, iucB, iucD, kpsD, kpsM, papA, papB, papI, papX, sat, senB, aggR, papE, papF* | *aadA5, acrS, aph(3'')-Ib, aph(6)-Id, bacA, baeR, baeS, bla-AmpC2, bla-AmpC1, bla-ampH, bla-CTX-M-3, bla-PBP, bla-TEM-1, bla-TEM-15, bla-TEM-1B, catII, cpxA, dfrA14, dfrA17, dhfr7, emrA, emrK, evgA, gadW, gadX, H-NS, marA, mdtA, mdtC, mdtE, mdtF, mdtG, mdtH, mdtM, oqxA, qnrS1, strA, strB, sul2, sul1, tet(A), tet(B), yojI* | *-* |

| **Isolate** | **Sample** | **Clade** | **Phylogroup** | **STs** | **O-type** | **H-type** | **Serotypes** | **Virulence Gene(s) - VGs** | **Antibiotic resistance gene(s) - ARGs** | **OAGC - IS** |
| --- | --- | --- | --- | --- | --- | --- | --- | --- | --- | --- |
| HS_08 | Human | B | B1 | 297 | O179 | H48 | O179:H48 | *aslA, cdiAB, cfaA, cfaB, cfaC, cfaD-E, csgA-B, ecpA-E, ehaA, ehaB, elfA-G, entA, entB, entC, espL1, espY, fdeC, fepA-C, fepD, fepG, fes, fimA, fimB-I, hcpA-C, hlyE, ibeB, ibeC, ompA, ompT, agn43, pic, cah* | *acrA, acrB, acrF, bacA, bla-AmpC1, bla-ampH, bla-PBP, bla-TEM-1, CRP, dfrA7, emrA, emrB, emrE, emrK, emrR, emrY, eptA, evgA, evgS, gadW, gadX, H-NS, marA, mdf(A), mdtC, mdtE, mdtH, mdtM, mdtP, mph(B), msbA, sat-2, sul2, sul1, tet(A), tet(B), tetR, tolC, ugd, yojI* | *IS5* |
| HS_09 | Human | A | A | 10 | O173 | H21 | O173:H21 | *aslA, cfaA, cfaB, cfaC, cfaD-E, csgA-B, ecpA-E, ehaB, elfA-G, entA, entB, entC, espL1, espY, fdeC, fepA-C, fepD, fepG, fes, fimA, fimB-I, hcpA-C, hlyE, ibeB, ibeC, ompA, ompT, agn43* | *acrA, acrE, acrF, acrS, ant(3''-IIa), aph(3'')-Ib, aph(6)-Id, baeR, bla-AmpC2, bla-AmpC1, bla-ampH, bla-CTX-M-3, bla-PBP, bla-TEM-1, bla-TEM-135, catA1, catB4, dfrA17, emrK, evgA, H-NS, marA, mdf(A), mdtC, mdtE, mdtF, mdtH, mdtM, mdtN, mdtO, strB, sul2, tet(A), tolC, ugd, yojI* | *IS5* |
| HS_10 | Human | A | A | 218 | O16 | H48 | O16:H48 | *aslA, csgA-B, ecpA-E, ehaB, elfA-G, entA, entB, entC, espL1, espY, fdeC, fepA-C, fepD, fepG, fes, fimA, fimB-I, hcpA-C, hlyE, ibeB, ibeC, ompA, ompT, tia, agn43, aggR* | *aac(6')-Ib, acrA, acrD, acrE, acrS, aph(3'')-Ib, aph(6)-Id, baeR, baeS, bla-AmpC2, bla-AmpC1, bla-ampH, bla-PBP, cpxA, CRP, dhfr7, emrA, emrE, emrK, emrR, eptA, evgA, evgS, gadW, kdpE, mdf(A), mdtA, mdtB, mdtC, mdtE, mdtG, mdtM, mdtN, mdtP, mph(B), msbA, pmrF, sul2* | *IS5* |

| **Isolate** | **Sample** | **Clade** | **Phylogroup** | **STs** | **O-type** | **H-type** | **Serotypes** | **Virulence Gene(s) - VGs** | **Antibiotic resistance gene(s) - ARGs** | **OAGC - IS** |
| --- | --- | --- | --- | --- | --- | --- | --- | --- | --- | --- |
| HS_11 | Human | B | B1 | 641 | O117 | H7 | O117:H7 | *aslA, csgA-B, ecpA-E, ehaA, ehaB, elfA-G, entA, entB, entC, espL1, espY, fdeC, fepA-C, fepD, fepG, fes, fimA, fimB-I, hcpA-C, hlyA, hlyC, hlyE, ibeB, ibeC, iha, ompA, ompT, afaA, afaC, afaD, agn43, fyuA, irp2, iucC, iutA, aafC, afaB, afaE, irpirp1, iucA, iucB, iucD, cdtA, cdtB, cdtC, cnfcnf1-2-2, tibA, stx, stx1, afaF* | *acrA, acrB, acrD, acrE, acrF, acrS, aph(3'')-Ib, aph(6)-Id, baeS, bla-AmpC2, bla-AmpC1, bla-ampH, bla-PBP, bla-TEM-1, bla-TEM-15, emrR, eptA, evgA, gadW, gadX, H-NS, marA, mdf(A), mdtA, mdtB, mdtC, mdtH, mdtO, strA, sul2, tet(A), tet(B), tolC* | *IS5* |
| HS_12 | Human | B | B1 | 3576 | O16 | H48 | O16:H48 | *aslA, cfaA, cfaB, cfaC, csgA-B, ecpA-E, ehaB, elfA-G, entA, entB, entC, espL1, espY, fdeC, fepA-C, fepD, fepG, fes, fimA, fimB-I, hcpA-C, hlyE, ibeB, ibeC, ompA, ompT, agn43, cah* | *acrA, acrB, acrD, acrE, acrF, acrS, bacA, baeR, baeS, bla-AmpC2, bla-ampH, bla-OXA, bla-TEM-15, catA1, cpxA, dfrA14, emrA, emrE, eptA, gadW, gadX, kdpE, marA, mdtB, mdtC, mdtF, mdtG, mdtN, mdtO, mdtP, mph(B), msbA, strA, strB, sul2, sul1, tet(A), tet(B), tolC, yojI* | *IS5* |
| HS_13 | Human | B | B1 | 1737 | O16 | H16 | O16:H16 | *aslA, cia, csgA-B, ecpA-E, ehaA, ehaB, elfA-G, entA, entB, entC, espL1, espY, fdeC, fepA-C, fepD, fepG, fes, fimA, fimB-I, hcpA-C, hlyA, hlyB, hlyC, hlyD, hlyE, ibeB, ibeC, iha, ompA, ompT, agn43, sitA-C, iucA, stx, stx1* | *acrS, bacA, baeS, bla-AmpC1, bla-ampH, bla-CTX-M-15, bla-TEM-1, catA1, cpxA, CRP, dfrA17, dhfr7, emrB, emrE, emrK, eptA, evgS, gadW, kdpE, mdf(A), mdtB, mdtC, mdtE, mdtH, mdtN, mdtO, mph(A), mph(B), msbA, pmrF, strB, sul2, tet(A), yojI* | *-* |
| HS_14 | Human | B | B1 | 58 | O117 | H48 | O117:H48 | *aslA, cfaA, cfaB, cfaC, cfaD-E, csgA-B, ecpA-E, ehaA, ehaB, elfA-G, entA, entB, entC, espL1, espY, fdeC, fepA-C, fepD, fepG, fes, fimA, fimB-I, hcpA-C, hlyE, ibeB, ibeC, ompA, ompT, agn43, tibA* | *acrA, acrB, acrD, aph(3'')-Ib, aph(6)-Id, bacA, baeR, bla-AmpC1, bla-ampH, bla-TEM-1, CRP, dfrA1, dfrA15, emrA, emrB, emrE, emrK, emrR, emrY, eptA, evgA, evgS, gadW, gadX, H-NS, kdpE, mdf(A), mdtB, mdtE, mdtF, mdtN, mph(B), pmrF, sul2, tet(B), tolC, ugd* | *IS5* |
| **Isolate** | **Sample** | **Clade** | **Phylogroup** | **STs** | **O-type** | **H-type** | **Serotypes** | **Virulence Gene(s) - VGs** | **Antibiotic resistance gene(s) - ARGs** | **OAGC - IS** |
| HS_15 | Human | B | B1 | 155 | O21 | H12 | O21:H12 | *aslA, csgA-B, ecpA-E, ehaA, ehaB, elfA-G, entA, entB, entC, espL1, espY, fdeC, fepA-C, fepD, fepG, fes, fimA, fimB-I, hcpA-C, hlyE, ibeB, ibeC, ompA, ompT, aatA, sat, aap/aspU, aggR, astA, SefB, sepA, vat* | *aadA5, acrA, acrD, acrE, acrF, acrS, aph(3'')-Ib, aph(6)-Id, bacA, baeR, bla-AmpC1, bla-TEM-1C, catA1, CRP, dfrA17, emrE, emrK, emrR, evgA, evgS, gadW, gadX, mdf(A), mdtA, mdtB, mdtC, mdtF, mdtM, mdtN, mdtO, mph(B), msbA, sul2, sul1, tet(A), tet(B), ugd, yojI* | IS5 |
| HS_16 | Human | B | B1 | 2350 | O16 | H48 | O16:H48 | *aslA, cfaA, cfaB, cfaC, cfaD-E, csgA-B, ecpA-E, ehaB, elfA-G, entA, entB, entC, espL1, espY, fepA-C, fepD, fepG, fes, fimA, fimB-I, hcpA-C, hlyE, ibeB, ibeC, ompA, ompT, agn43* | *aac(3)-IIa, aac(6')-Ib, acrB, aph(3'')-Ib, bacA, baeR, baeS, bla-AmpC2, bla-AmpC1, bla-ampH, bla-PBP, bla-TEM-1B, catA1, cpxA, dfrA27, dfrA7, emrA, emrB, emrE, emrR, emrY, eptA, evgS, gadX, mdf(A), mdtA, mdtC, mdtE, mdtF, mdtG, mdtH, mdtM, mdtN, mdtP, mph(B), msbA, sul2, sul1, tet(A), ugd, yojI* | *IS5* |
| HS_17 | Human | C | Unknown | 1193 | O16 | H48 | O16:H48 | *aslA, cfaA, cia, csgA-B, ecpA-E, elfA-G, entA, entB, entC, espL1, espY, fdeC, fepA-C, fepD, fepG, fes, fimA, fimB-I, hcpA-C, hlyE, ibeB, ibeC, iha, ompA, ompT, agn43, fyuA, irp2, iucC, iutA, sitA-C, chuA, chuS, chuT, chuU, chuV, chuW, chuX, chuY, irpirp1, iucA, iucB, iucD, kpsD, kpsM, papA, papB, papI, papX, sat, senB, kpsT, vat, tsh, upaH* | *acrB, acrD, acrF, acrS, aph(3'')-Ib, aph(6)-Id, bacA, bla-AmpC2, bla-AmpC1, bla-ampH, bla-TEM-1, bla-TEM-15, bla-TEM-1B, bla-TEM-1D, cpxA, dfrA5, emrE, emrK, emrR, emrY, evgA, evgS, fosA, gadX, H-NS, mdf(A), mdtA, mdtE, mdtF, mdtG, mph(B), qnrS1, strB, sul1, tet(A), tet(B), tetR, ugd, yojI* | *IS5* |

| **Isolate** | **Sample** | **Clade** | **Phylogroup** | **STs** | **O-type** | **H-type** | **Serotypes** | **Virulence Gene(s) - VGs** | **Antibiotic resistance gene(s) - ARGs** | **OAGC - IS** |
| --- | --- | --- | --- | --- | --- | --- | --- | --- | --- | --- |
| HS_18 | Human | C | E | 69 | O15 | H48 | O15:H48 | *aslA, csgA-B, ecpA-E, elfA-G, entA, entB, entC, espL1, espY, fdeC, fepA-C, fepD, fepG, fes, fimA, fimB-I, hcpA-C, hlyA, hlyB, hlyC, hlyD, hlyE, ibeB, ibeC, iha, ompA, ompT, tia, fyuA, irp2, sitA-C, chuA, chuS, chuT, chuU, chuV, chuW, chuX, chuY, irpirp1, kpsM, papA, papB, papI, sat, shuA, aggR, astA, shuT, iroN, papC, iroB, iroC, iroD, iroE, ireA, papD, papE, papF, papG, papH, papJ, papK* | *acrF, acrS, aph(3'')-Ib, baeR, baeS, bla-AmpC2, bla-ampH, bla-OXA, bla-PBP, bla-TEM-1, bla-TEM-15, cpxA, CRP, dfrA7, emrA, emrB, emrE, emrK, emrR, H-NS, marA, mdf(A), mdtA, mdtB, mdtC, mdtF, mdtH, mdtM, mdtN, mdtO, pmrF, sul2, sul1, tet(A), tet(B), tet(D), tetR, ugd, yojI* | - |
| HS_19 | Human | A | A | 1703 | O15 | H48 | O15:H48 | *aslA, csgA-B, ecpA-E, ehaB, elfA-G, entA, entB, entC, espL1, espY, fdeC, fepA-C, fepD, fepG, fes, fimA, fimB-I, hcpA-C, hlyE, ibeB, ibeC, ompA, ompT, aatA, agn43, sat, aap/aspU, aggR, astA, SefB, sepA, vat* | *acrA, acrF, aph(3'')-Ib, aph(6)-Id, arr-3, bacA, baeS, bla-AmpC1, bla-ampH, bla-CTX-M-3, bla-TEM-1B, catA1, cpxA, CRP, dfrA27, dfrA5, fosA6, gadX, H-NS, mdf(A), mdtB, mdtE, mdtF, mdtH, mdtM, mdtP, msbA, strA, sul2, sul1, tet(A), tet(B), tolC, ugd, yojI* | IS5 |
| HS_20 | Human | A | A | 1122 | O16 | H48 | O16:H48 | *aslA, csgA-B, ecpA-E, ehaB, elfA-G, entA, entB, entC, espL1, espY, fdeC, fepA-C, fepD, fepG, fes, fimA, fimB-I, hcpA-C, hlyE, ibeB, ibeC, ompA, ompT, stx2, agn43, stx, stx1* | *aadA1, acrF, acrS, aph(6)-Id, bacA, baeR, baeS, bla-AmpC1, bla-ampH, bla-PBP, bla-TEM-1, bla-TEM-15, bla-TEM-1B, cpxA, CRP, dfrA7, dfrA14, emrA, evgA, evgS, kdpE, mdf(A), mdtA, mdtB, mdtF, mdtG, mdtM, mdtP, msbA, pmrF, strB, sul2, tet(A), tet(B), tetR, tolC, ugd, yojI* | IS5 |

| **Isolate** | **Sample** | **Clade** | **Phylogroup** | **STs** | **O-type** | **H-type** | **Serotypes** | **Virulence Gene(s) - VGs** | **Antibiotic resistance gene(s) - ARGs** | **OAGC - IS** |
| --- | --- | --- | --- | --- | --- | --- | --- | --- | --- | --- |
| HS_21 | Human | B | B1 | 205 | O100 | H48 | O100:H48 | *aslA, cfaA, cfaB, cfaC, cfaD-E, csgA-B, ecpA-E, ehaA, elfA-G, entA, entB, entC, espL1, espY, fdeC, fepA-C, fepD, fepG, fes, fimA, fimB-I, hcpA-C, hlyE, ibeB, ibeC, ompA, ompT, cah* | *acrB, acrD, acrS, aph(3'')-Ib, aph(6)-Id, baeR, baeS, bla-AmpC1, bla-ampH, bla-PBP, bla-TEM-1B, cpxA, CRP, dfrA1, dhfr7, emrA, emrB, emrE, emrY, evgA, gadW, gadX, H-NS, mdf(A), mdtA, mdtB, mdtC, mdtE, mdtF, mdtG, mdtH, mdtM, mdtP, sul2, tet(A), tolC, ugd, yojI* | *IS5* |
| HS_22 | Human | B | B1 | 869 | O16 | H48 | O16:H48 | *aslA, cfaA, cfaB, cfaC, cfaD-E, csgA-B, ecpA-E, ehaA, ehaB, elfA-G, entA, entB, entC, espL1, espY, fdeC, fepA-C, fepD, fepG, fes, fimA, fimB-I, hcpA-C, hlyE, ibeB, ibeC, ompA, ompT, agn43, eltA* | *acrA, aph(3'')-Ib, aph(6)-Id, bacA, baeR, baeS, bla-AmpC1, bla-ampH, bla-CTX-M-3, bla-PBP, bla-TEM-1, bla-TEM-1B, catA1, dfrA5, dfrA7, emrB, emrE, emrK, eptA, gadX, kdpE, mdf(A), mdtB, mdtC, mdtE, mdtF, mdtM, mdtO, mdtP, msbA, sul2, tet(A), tet(B), ugd* | *IS5* |
| HS_23 | Human | A | A | 10 | O13 | H48 | O13:H48 | *aslA, cdiAB, csgA-B, ecpA-E, ehaB, elfA-G, entA, entB, entC, espL1, espY, fepA-C, fepD, fepG, fes, fimA, fimB-I, hcpA-C, hlyE, ibeB, ibeC, ompA, ompT, stx2, tia, agn43* | *acrA, acrB, acrD, acrE, acrF, acrS, aph(3'')-Ib, baeR, bla-AmpC2, bla-AmpC1, bla-ampH, bla-PBP, bla-TEM-1, bla-TEM-1B, cpxA, CRP, dfrA7, emrE, emrY, eptA, evgS, gadX, marA, mdf(A), mdtA, mdtB, mdtF, mdtG, mdtH, mdtO, mdtP, msbA, sul1, tolC, ugd, yojI* | *IS5* |
| HS_24 | Human | B | B1 | 3576 | O153 | H48 | 0153:H48 | *aslA, cfaA, cfaB, cfaC, csgA-B, ecpA-E, ehaA, ehaB, elfA-G, entA, entB, entC, espL1, espY, fdeC, fepA-C, fepD, fepG, fes, fimA, fimB-I, hcpA-C, hlyA, hlyC, hlyE, ibeB, ibeC, iha, ompA, ompT, afaA, afaC, afaD, agn43, eltA, iucC, iutA, aafC, afaB, afaE, iucA, iucB, iucD, cdtA, cdtB, cdtC, cnfcnf1-2-2, tibA, afaF* | *acrA, acrB, acrE, acrF, aph(3'')-Ib, aph(6)-Id, bacA, baeR, baeS, bla-AmpC2, bla-AmpC1, bla-ampH, bla-PBP, bla-TEM-15, bla-TEM-1B, catI, cpxA, emrA, emrR, eptA, evgA, evgS, gadW, gadX, H-NS, kdpE, marA, mdtB, mdtC, mdtE, mdtF, mdtP, mph(B), msbA, pmrF, strB, sul2, sul1, tet(A), tetR, yojI* | *IS5* |
| **OAGC – IS – represents O-Antigen Gene cluster contained Insertion sequnce** | | | | | | | | | | |
